# The Coevolution of Colour Patterns and Hindwing Shapes on a Large Phylogenetic Scale Reveals Predation‐Driven Adaptive Syndromes in Swallowtail Butterflies

**DOI:** 10.1111/ele.70303

**Published:** 2026-01-18

**Authors:** Agathe Puissant, Ariane Chotard, Fabien L. Condamine, Vincent Debat, Violaine Llaurens

**Affiliations:** ^1^ Institut de Systématique, Evolution et Biodiversité (UMR 7205 CNRS/MNHN/SU/EPHE/UA), Muséum National d'Histoire Naturelle ‐ CP50 Paris France; ^2^ Centre Interdisciplinaire de Recherche en Biologie (UMR 7241 CNRS/INSERM/Collège de France) Paris France; ^3^ Institut des Sciences de l'Evolution de Montpellier (Université de Montpellier/CNRS/IRD) Montpellier France

**Keywords:** colour pattern, developmental constraints, geometric morphometrics, machine learning, papilionidae, predation deflection

## Abstract

Traits that reduce predation success may evolve together, leading to repeated evolution of similar anti‐predator syndromes. In butterflies, predation likely shapes wing shape and colour patterns, promoting either aposematic or deflective features. Here, we studied the evolution of hindwing tail shape and colour pattern across swallowtail butterflies. Using standardised museum specimen photographs, we quantified colour variation via computer vision and tail shape with geometric morphometrics. We found significant evolutionary correlations between colour patterns and hindwing tail shapes across the phylogeny. Long tails were linked to high‐contrast stripes and marginal spots, while short tails were associated with simple, spotted patterns. While accounting for developmental constraints, we show that stripes, spots, and long tails evolved in correlation and likely form a visual syndrome promoted by natural selection to deflect predator attacks. These results provide evidence that selection can drive the coordinated evolution of complex anti‐predator traits over large evolutionary timescales.

## Graphical abstract

By combining computer vision and morphometrics on museum specimens, we show that hindwing tail shape and colour patterns evolved in concert across swallowtails. Long‐tailed species display contrasted stripes and marginal spots, suggesting coevolution of deflective traits promoted by natural selection by predators.

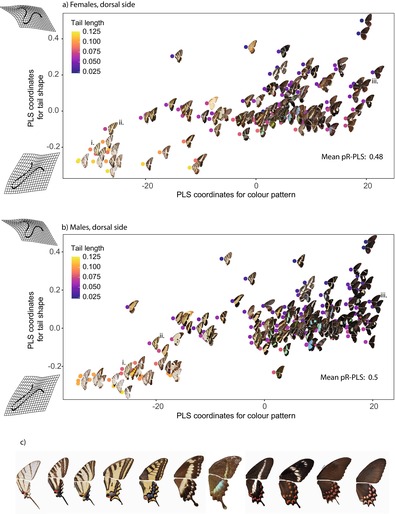

## 1 Introduction

Specific trait combinations can be repeatedly observed in different species occupying similar ecological niches. For example, ecomorphs in *Anolis* lizards adapted to similar habitats exhibit consistent morphological traits across islands: twig‐dwelling species display short limbs and tails facilitating movement on narrow surfaces, while trunk‐ground dwellers have longer limbs and large toe pads aiding clinging (Elstrott and Irschick 2004). The coordinated evolution of phenotypic traits under specific selective pressure can result in adaptive syndromes (Sinnott‐Armstrong et al. 2022). However, the repeated emergence of identical phenotypic combinations across lineages raises fundamental questions about the developmental and selective constraints underlying trait evolution within a given syndrome. Macroevolutionary investigations of such trait suites remain scarce but are crucial for understanding the underlying evolutionary processes.

Trait associations observed among species often result from neutral processes: species inherit trait suites from common ancestors, leading closely related species to display similar phenotypes (Losos 2008). Correlations may also reflect shared developmental constraints. Traits shaped by interacting developmental mechanisms are likely to remain integrated at the evolutionary scale (Felice et al. 2018; Klingenberg 2008). Similarly, genetic correlations—due to pleiotropy or linkage—may drive trait coevolution (Saltz et al. 2017). Correlated selection can reinforce or shape such genetic linkages (Sinervo and Svensson 2002; Svensson et al. 2021), while selection may eliminate deleterious pleiotropic effects (Pavlicev and Wagner 2012). Thus, both correlated selection and genetic correlation jointly affect phenotypic trait evolution. Disentangling adaptive syndromes from correlations stemming from other processes is challenging, requiring phylogenetic analyses to investigate joint evolutionary history of traits, as well as supporting evidence for shared selective pressures.

Prey–predator interactions strongly drive the joint evolution of multiple phenotypic traits across species (Langerhans 2007; Langerhans et al. 2004). Specific combinations of behavioural and/or morphological traits may enhance survival by reducing predation risk or improving post‐attack survival. Such traits may be co‐selected across prey species. Predation can drive the evolution of traits that lead to cryptic phenotypes such as chironomid larvae that display a motionless behaviour that acts synergistically with background‐matching colour, jointly decreasing detection (Ioannou and Krause 2009). Conversely, predators may learn to avoid prey bearing aposematic traits linked to unpalatability (Caro and Ruxton 2019), favouring the evolution of suites of visually perceived traits enhancing prey recognition. Similarly, deflective syndromes—redirecting predator attacks to non‐vital body parts (Caro and Allen 2017; Humphreys and Ruxton 2018)—often combine morphological, physiological, and behavioural traits. In lizards, tail autotomy and conspicuous tail colouration co‐evolved (Murali and Kodandaramaiah 2016), sometimes with tail waving (e.g., Telemeco et al. 2011). Thus, the evolution of cryptic, aposematic, and deflective syndromes in prey species usually entails the evolution of complex trait combinations because of selective pressures generated by (1) predator detection capacities and attack behaviour, (2) the composition of alternative palatable and unpalatable prey communities for aposematic and deflective syndromes, or (3) the complexity of the background used by cryptic prey.

Predation‐driven evolution depends on predator sensory systems (Ratcliffe and Nydam 2008; Roberts et al. 2007). In nocturnal luna moths, long, twisted hindwing tails deflect echolocating bats by altering sonar reflections (Barber et al. 2015). Visual predators also impose strong selection on prey colour patterns (Bond and Kamil 2002), along with morphology and behaviour (Forsman and Appelqvist 1998). Some studies suggest that appendage elongation coupled with striped patterns may help deflect attacks (Jackson et al. 1976; Murali and Kodandaramaiah 2016; Urban et al. 2022). Predation experiments in lizards revealed that striped tails enhanced deflection (Murali and Kodandaramaiah 2016), while a comparative study in reef fish linked body elongation with longitudinal stripes (Urban et al. 2022). Such striped patterns can create a motion dazzling effect—confusing predator perception of speed and direction (Stevens 2007)—and shift attacks rearwards (Murali and Kodandaramaiah 2016).

In Lepidoptera, several lineages independently evolved elongated hindwings or tails (Barber et al. 2015; Owens et al. 2020), which vary in number and shape (Hendrick et al. 2022). Forewing shape affects flight performance (Le Roy et al. 2019), and hindwing shape, including tails, may influence gliding stability (Park et al. 2010). Hindwing morphology and colour patterns together impact butterfly appearance. Predation‐driven selection may shape these features: for instance, polymorphic mimicry in some species matches local unpalatable models. For instance, in *Papilio memnon* (now *P. agenor*), one female morph has spatulate tails, similar to the sympatric species *Pachliopta aristolochiae*, while the non‐mimic form is tailless (Clarke et al. 1997). Conversely, in *Papilio dardanus*, tailed males contrast with tailless mimetic females resembling unpalatable Danainae models (Nijhout 2003). These striking tail differences within polymorphic species likely reflect predator‐mediated interactions and the local composition of unpalatable prey species.

Alternatively, hindwing tail evolution may reflect selection for attack deflection. In Lycaenidae butterflies, hindwing tails mimic antennae and combine with conspicuous spots and a hindwing‐rubbing behaviour in a “false‐head” syndrome (Hendrick et al. 2022; Robbins 1981). Experiments on *Calycopis cecrops* showed that jumping spiders preferentially attacked the false‐head, a non‐vital part of the body (Sourakov 2013). Similarly, in Papilionidae, some species display tails with marginally placed coloured dots contrasting with black‐and‐white striped patterns. Behavioural experiments using great tits attacking *Iphiclides podalirius* models revealed a bias towards the tail area (Chotard et al. 2022). While debate continues over the relative role of tails vs. marginal spots in deflecting attacks (Kodandaramaiah 2011), even tailless species like *Lopinga achinae* show that marginal spots can deflect avian attacks (Olofsson et al. 2010). These findings suggest that the evolution of these colour pattern features can evolve independently of tails to divert attacks. However, the sequence of trait evolution leading to these visual syndromes remains unresolved.

In butterflies, colour pattern elements such as spots likely develop through morphogen diffusion from foci within wing cells, bounded by veins (Nijhout 1978, 2001). Given the presumed conservation of this developmental system, one can predict element positions based on wing venation (Mazo‐Vargas et al. 2024; Nijhout 2001). Wing shape changes, such as tail elongation, can stretch neighbouring cells and shift morphogen foci distally, altering colour element positions. Thus, tail elongation may influence colour pattern layout. Developmental constraints should therefore be considered when interpreting pattern‐shape associations. In addition, the expression of colour pattern genes is spatially restricted by veins, which can produce pattern discontinuities—spots expressed in some regions but not others (McKenna et al. 2020). A survey across Lepidoptera showed these discontinuities often occur near hindwing tails (McKenna et al. 2020). Therefore, wing shape variation might be correlated with the presence or absence of colour elements in different wing regions.

Here, we investigate the evolution of hindwing tail shape and colour pattern elements in Papilionidae butterflies to shed light on how visual traits evolved under selection. These day‐flying butterflies show a wide range of aposematic and mimetic wing patterns (Kunte 2009; Puissant et al. 2023), along with substantial hindwing variation including tails differing in size and shape (Owens et al. 2020). We aim at testing for correlated evolution between hindwing colour pattern and tail shapes across Papilionidae. We further identify conspicuous pattern elements associated with tail evolution, aiming to characterise the repeated emergence of visual syndromes during the diversification of Papilionidae.

## 2 Methods

### 2.1 Standardised Photography of Museum Specimens and Machine‐Learning Quantification of Whole Colour Pattern Variation

We sampled 1358 specimens from the National Museum of Natural History (Paris), spanning 329 species for males and 273 species for females. Photographs were taken using a Nikon D90 (Camera lens: AF‐S Micro Nikkor 60 mm 1:2.8G ED) in standardised conditions, in a dark room with controlled LED lighting and diffusers. All pictures were taken at 90° of the planes of the wings. Both dorsal and ventral sides were photographed, with 2–5 specimens per sex and species when available, and additional sampling for polymorphic species.

To quantify whole colour pattern variation among species, we used the deep learning SimCLR method (Chen et al. 2020) which enables homology‐free comparison across distantly related species varying in shape and colour patterns (see Puissant et al. 2023 for more detail). SimCLR is a deep metric learning approach based on convolutional neural networks. The network is trained to quantify image similarity using unsupervised feature extraction, avoiding human classification bias. We trained the neural network with augmentations chosen to reduce reliance on wing shape features. As tails are a striking feature of many butterflies, we removed the pixels contained within the hindwing tail landmarks from the images prior to training to limit the contribution of wing shape variation to the ML embedding (see method details in Data S1: Section 1a and Puissant et al. 2023 for more details on training). We then performed a PCA on the machine learning embeddings, retaining the first 24 dimensions, each accounting for at least 1% of the variance, collectively explaining 77.2% of the total variance.

### 2.2 Geometric Morphometrics of Hindwing Tail Shape

We quantified hindwing shape using geometric morphometrics with 19 fixed landmarks at vein intersection and termini, and 145 semi‐landmarks on wing outlines (Figure 3a), superimposed via generalised Procrustes analysis (Rohlf and Slice 1990, see method details in Data S1: Section 1b. For more details on landmark processing). To specifically study the evolution of tails, we focused on the landmarks located between veins M2 and CU1 (1 fixed landmark and 50 semi‐landmarks) as the tails of *Papilionidae* in our dataset are always localised in this area (Figure 3a).

To find and visualise the main clusters of wing shape based on these landmarks, we used adaptive spectral clustering, which is suitable for high dimensional data (*Spectrum* R package, John et al. 2020) separately on males and females, using the eigengap method and setting the maximum number of clusters to 5.

### 2.3 Correlation Between Tail Shape and Colour Pattern

We investigated the correlation between colour pattern and hindwing tail shape at two levels. First, we included all species, that is, tailed and tailless ones, to assess the association of the presence/absence of tail and colour pattern in the whole dataset. Second, we focused exclusively on tailed species to finely quantify the link between tail shape and colour pattern.

Covariation between shape and colour pattern was assessed by phylogenetic two‐block partial least square analysis (pPLS) using the *phylo.integration* function from the R package *geomorph* (Adams and Otárola‐Castillo 2013). We used the 24‐dimensional colour pattern coordinates and the 102‐dimensional shape coordinates, respectively as colour pattern and shape descriptors. For polymorphic species, we averaged coordinates within each form. The complete dataset comprised 267 species (288 phenotypic forms) for females and 324 species (324 forms) for males, while the sub‐dataset with species exhibiting tails comprised 116 species (118 phenotypic forms) for females and 149 species (149 forms) for males. We assumed the phylogenetic relationships reconstructed by Allio et al. (2021). We found that a lambda model of evolution best fitted our data using the *mvMorph* package (Clavel et al. 2015), and thus lambda‐transformed the phylogenetic tree before running the analyses. To account for intraspecific variation of colour pattern and shape, we randomly sampled one individual per species and recorded the phylogenetic R‐PLS (pR‐PLS) when significant at the 5% threshold. We repeated this process 1000 times and obtained a distribution of pR‐PLS describing the correlation. Because the correlation between tail and colour can vary between sex and wing side, these analyses were performed separately for each sex and wing side, using both the whole dataset and the sub‐dataset with tailed species only.

### 2.4 Effect of Ecological Variables on Tail Shape/Colour Pattern Associations

We tested for the effect of ecological variables on tail shape/colour pattern association by extracting species coordinates on the axes of variation of the two block PLS. We then performed a multivariate phylogenetic regression on these coordinates using the *mvgls* function from the *mvMorph* package and assessed significance using the *manova.gls* function. We used two variables as predictors: (1) main host‐plant to test for an effect of unpalatability, and (2) main biome to test for an effect of environment (data acquisition detailed in method details Data S1: Section 1c).

### 2.5 Correlation Between Rates of Colour Pattern and Tail Shape Evolution

We computed multivariate rates of evolution for colour pattern and tail shape separately using the *RRphylo* package (Castiglione et al. 2018) for each sex and wing side. As for the pR‐PLS calculation, we accounted for intraspecific variation by sampling one random specimen per species, computing the branch‐wise rates of evolution, and repeating this process 100 times. For each sampling, we fitted a linear model between estimated branch rates for colour pattern and for shape and computed the *R* ^2^ to obtain a distribution of *R* ^2^.

### 2.6 Colour Pattern Descriptors

#### 2.6.1 Granularity Analysis to Describe Broad Characteristics of the Colour Pattern

To identify the colour pattern features specifically associated with tail shape variation, we computed several colour pattern descriptors using the granularity analysis from MicaToolbox (van den Berg et al. 2020). We used images of the butterflies with the hindwing tails removed. These images are processed via a series of bandpass filters, using the difference of Gaussians method, which quantifies visual texture at different spatial scales. From this analysis, we extracted three quantities describing (1) the contrast level of the dominant markings, that is, the most visually prominent elements of the colour pattern, defined as the spatial scale with the strongest filter response (2) the size of these dominant markings, and (3) the overall complexity of the colour pattern (see method details in Data S1: Section 1d for details).

#### 2.6.2 Detection of Wing Spots

To investigate the correlation between tail shape and specific conspicuous elements of colour patterns, we developed custom image analysis code in R and Python to detect contrasting circular spots on the hindwings (for more details see method details in Data S1: Section 1e and extract several measures from these spots). All operations and parameters are provided in the Python code available at: https://github.com/AgathePuissant/Correlation_tail_colour_evolution. We then visualised the localisation of detected spots on the main modalities of wing shape, shown in the density plots (Figure 3b for the dorsal side of males and Figure S1).

For each detected spot, we fitted an ellipse around the spot and extracted (1) the distance from the center of the ellipse to the closest wing contour point, corresponding to the minimum distance from the spot to the wing contour and (2) the distance from the center of the ellipse to the fixed landmark CU2 (tail base) (Figure 3a). We also calculated (3) the distance from the center of the ellipse to the centroid of the wing cell delimited by wing venation, corrected by the aspect ratio (elongation) of the cell. This represents a measure of departure of the spot from the center of the wing cell.

The centroid of the wing cell was considered as the position of the foci of the spot/eyespot, assuming a classical developmental pathway with foci of morphogen diffusion constrained within each wing cell at the midline of the cell (Mazo‐Vargas et al. 2024; Nijhout 1978, 2001). Computing the departure from this central position allows estimating how much the variation in the position of the spots is driven by physical shape variation of wing cells. Spots found more distant from wing cell centroids than others are thus assumed to result from an overcome of the developmental constraints applied by wing shape and venation.

As hindwing elongation into tails likely displaces the morphogen foci towards the wing outlines by physical distortion of the wing cells, we expect that spots will tend to be closer to the contours in tailed species only due to developmental constraints. However, a stronger departure of spot position from wing cell centroids in those species could indicate that the spot position may be influenced not only by physical developmental constraints but also by additional factors such as selective pressures. All measures were divided by wing area to standardise distances across species with different sizes.

We digitally removed the tails from the images before the quantification of colour pattern variation with machine learning and with traditional computer vision (granularity and spots analyses) to avoid spurious correlation between tail shape and colour stemming from bleed‐through of shape information into colour pattern quantification. However, some species display some patterning on the hindwing tails such as coloured spots. We assessed the impact of removing the tails on the quantification of colour pattern variation and found little impact (see more detailed discussion in Data S1: Sections 5 and 6).

### 2.7 Phylogenetic Regression on Tail Shape

We performed a PCA on tail shape, on both the whole dataset, including untailed species, and the sub‐dataset focusing on tailed species only. We focused on the first axis of variation for both PCAs, which explained 92% of variance for both females and males for the complete dataset: this first axis primarily explained the variation in tail presence. For the sub‐dataset of tailed species, the first axis of the PCA explained 65% and 62% of variance for females and males respectively and primarily described the variation from spatulated tails to thin, long tails (Tables 1 and 2). Performing two separate analyses on the PC axes from the complete dataset and the tailed species sub‐dataset allows capturing variation at different scales, highlighting (1) differences associated with tail presence vs. absence and (2) more subtle shape variation across species with hindwing tails, respectively.

**TABLE 1:** Phylogenetic regressions indicate that tail elongation is associated with spots closer to the contours and to the CU2 landmarks. P‐values inferior to 0.05 are in bold.

Term	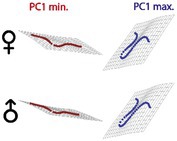		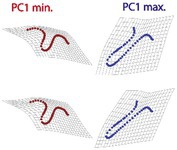	
	All species		Only tailed species	
	Estimate	*p*	Estimate	*p*
Females, dorsal side				
Distance to contours	**−0.051**	**0.005**	−0.030	0.086
Distance to CU2	−0.036	0.070	**−0.064**	**0.013**
Distance to contours:Distance to CU2			−0.022	0.115
Females, ventral side				
Distance to contours	−0.007	0.697	−0.030	0.101
Distance to CU2	**−0.062**	**0.004**	**−0.044**	**0.038**
Distance to centroids	0.018	0.371		
Distance to contours:Distance to centroids	**0.030**	**0.036**		
Males, dorsal side				
Distance to contours	**−0.052**	**0.007**	−0.022	0.118
Distance to CU2	**−0.044**	**0.019**	**−0.043**	**0.027**
Distance to centroids			−0.006	0.658
Distance to contours:Distance to CU2			−0.023	0.087
Distance to CU2:Distance to centroids			0.017	0.154
Males, ventral side				
Distance to contours	**−0.040**	**0.027**	−0.013	0.328
Distance to CU2	**−0.054**	**0.006**	−0.026	0.097
Distance to centroids	0.028	0.141	−0.011	0.331
Distance to contours:Distance to centroids	**0.028**	**0.038**	0.013	0.085

*Note:* On the top row: deformation grid from the mean shape to the extreme negative (red) and positive (blue) values of the first axis of tail shape variation for females on top and males on the bottom. Red colour depicts negative associations with tail elongation, blue colour depicts positive association with tail elongation. *p*‐values inferior to 0.05 are in bold.

**TABLE 2:** Phylogenetic regressions indicate that tail elongation is associated with finer markings and contrasted colour patterns.

Term	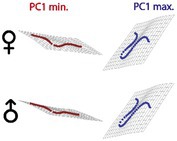		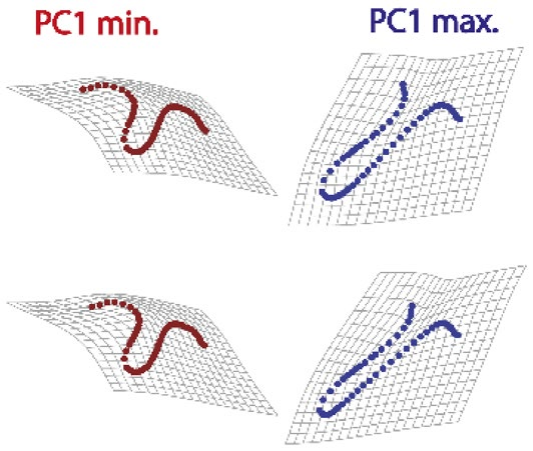	
	All species		Only tailed species	
	Estimate	*p*	Estimate	*p*
Female, dorsal side				
Marking size	**−0.043**	**0.025**	**−0.032**	**0.028**
Marking contrast			0.032	0.100
Marking dominance			−0.003	0.884
Marking size:Marking contrast			**0.061**	**0.002**
Marking size:Marking dominance			**−0.038**	**0.018**
Female, ventral side				
Marking size			−0.005	0.727
Marking contrast			**0.047**	**0.025**
Marking dominance			−0.025	0.216
Marking size:Marking contrast			**0.077**	**0.001**
Marking size:Marking dominance			**−0.056**	**0.004**
Male, dorsal side				
Marking size	**−0.050**	**0.004**	−0.012	0.262
Marking contrast	**0.060**	**0.004**	0.016	0.261
Marking dominance			−0.017	0.208
Marking size:Marking contrast			**0.051**	**0.003**
Marking size:Marking dominance			**−0.029**	**0.012**
Male, ventral side				
Marking size	**−0.061**	**0.001**	**−0.025**	**0.030**
Marking contrast	**0.094**	**0.002**	0.025	0.080
Marking dominance	−0.005	0.800	−0.016	0.247
Marking size:Marking contrast	**0.065**	**0.009**	**0.036**	**0.006**
Marking size:Marking dominance	**−0.055**	**0.003**	**−0.035**	**0.007**
Marking contrast:Marking dominance	0.020	0.111		

*Note:* On the top row: deformation grid from the mean shape to the extreme negative (red) and positive (blue) values of the first axis of tail shape variation for females on top and males on the bottom. Red colour depicts negative associations with tail elongation, blue colour depicts positive association with tail elongation. *p*‐values inferior to 0.05 are in bold.

We then performed phylogenetic regression on those PC1 axes using the *phyloglm* function from the *phylolm* R package (Ho et al. 2024), while jointly estimating *lambda* values of the phylogenetic signal. First, we performed a phylogenetic regression between PC1 axes and spots measurements on species that displayed spots. Then, we performed a second phylogenetic regression between PC1 axes and granularity analysis measurements. All predictors were z‐normalised prior to regression. For each analysis, we included all predictors and their interactions, using a stepwise approach to select the best fitting model using the AIC criterion computed with the *phylostep* function from the *phylolm* R package. When we detected a significant allometric effect, we corrected the analyses for hindwing size using the hindwing area with tails removed as a fixed effect.

## 3 Results

### 3.1 Correlated Evolution of Hindwing Tail Shape and Colour Pattern

**FIGURE 1:**
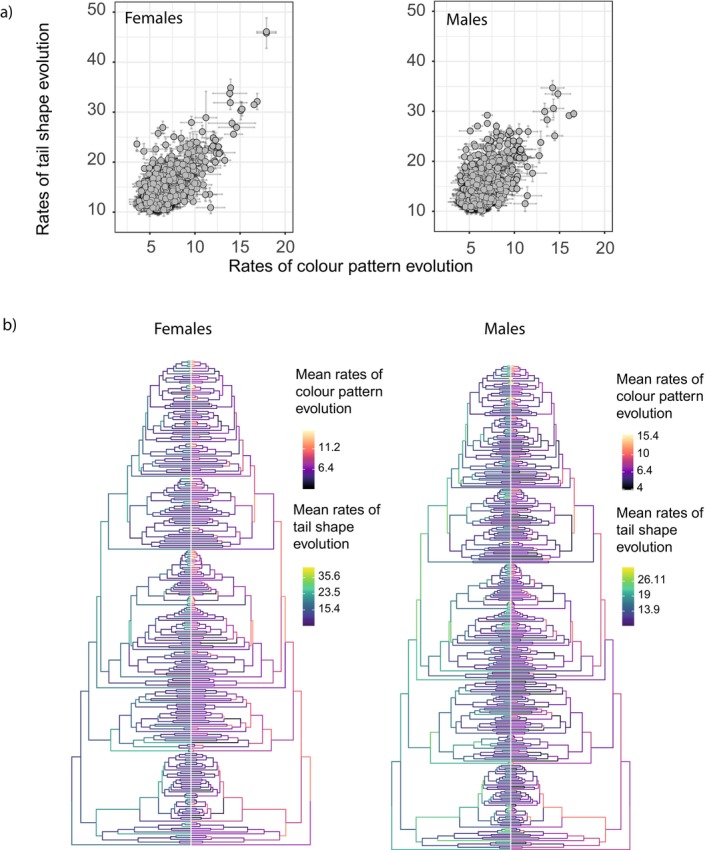
Correlated multivariate rates of tail shape and dorsal colour pattern evolution along the phylogeny. (a) Mean branch rates of dorsal colour pattern evolution vs. mean branch rates of tail shape evolution with the standard deviation of the 100 intraspecific sampling as error bars. (b) Mean branch rates of tail shape and dorsal colour pattern for the 100 intraspecific sampling mapped onto the phylogeny.

We investigated the tempo of evolution of hindwing tail shape and colour pattern to test for putative joint evolution. We found consistent significant positive correlations (*p* < 0.05 for all 100 intraspecific samplings) of rates of colour pattern evolution and rates of shape evolution (Figure 1 for the dorsal sides; Figure S2 for the ventral sides), with a mean *R* ^2^ of 0.4 and 0.41 for the dorsal and the ventral sides of females, and 0.27 and 0.36 for the dorsal and the ventral sides of males, respectively. These correlations in evolutionary rates reinforce the signal of coevolution of tail shape and colour pattern.

We then investigated the correlation between wing shape and the whole colour pattern. When analysing all species together, we found significant associations (*p* < 0.05 for all pPLS) between colour pattern and wing shape, with pR‐PLS ranging from 0.51 to 0.62 (Table S2). These associations were strongly driven by the large shape difference between untailed vs. tailed hindwings (Figure S3).

**FIGURE 2:**
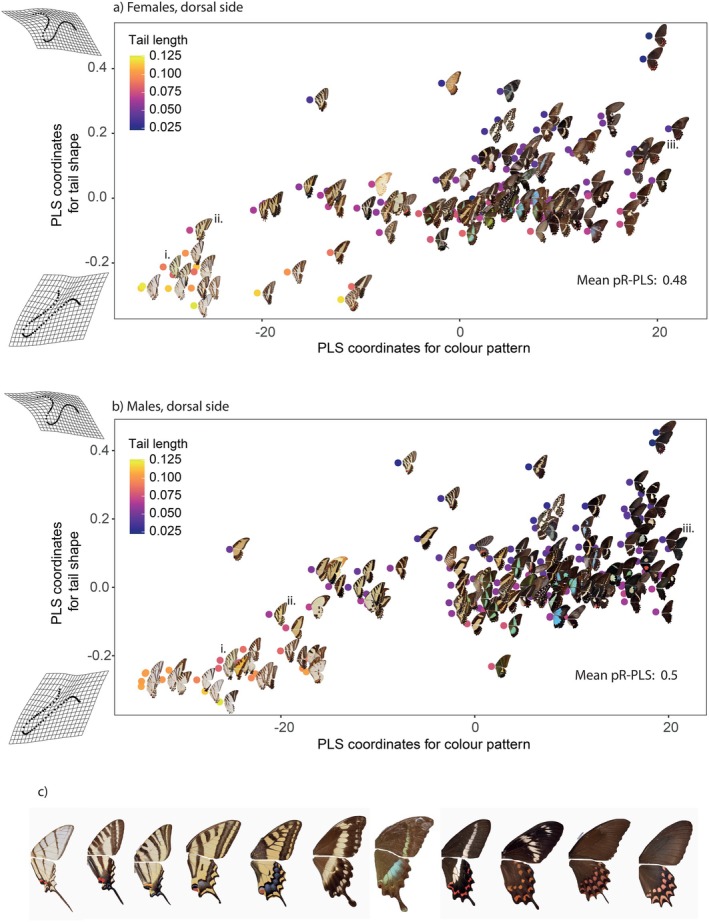
Significant associations detected between tail shape and tail‐removed dorsal wing colour pattern in tailed species, showing that longer tails are associated with striped patterns. Example pictures of specimens with tails removed are mapped on two‐block PLS species coordinates. (a) Two‐block PLS projection between tail shape and colour pattern for females (mean *pR‐PLS* = 0.48, *p < 0.05* for all 1000 intraspecific re‐samplings) i: *Iphiclides podalirius*, ii: *Papilio alexanor*, iii: *Papilio erostratus*, (b) Two‐block PLS projection between tail shape and colour pattern for males (mean *pR‐PLS* = 0.50, *p < 0.05* for all 1000 intraspecific re‐samplings). (c) Continuum of tail shape and colour pattern from striped species associated with thin tails, and spotted species associated with shorter, round tails. Pictures are displayed for the dorsal sides of females and are ordered based on the coordinates from the two block‐PLS between tail shape and colour patterns in tailed species. From left to right: *Protographium molops*, *Protographium marcellus*, *Iphiclides podalirius*, *Papilio alexanor*, *Papilio machaon* , *Papilio phorcas*, *Papilio crino*, *Parides bunichus*, *Pachliopta hector*, *Papilio erostratus*, *Parides photinus*.

When focusing on species with tailed hindwings only, we found significant associations in the 2B‐PLS, with R‐PLS ranging from 0.48 to 0.5 (*p* < 0.05 for all pPLS, Table S2). These associations were mainly driven by the difference between species displaying thin and long tails, such as *Iphiclides podalirius* vs. species displaying shorter tails, such as *Papilio erostratus* (Figure 2 for the dorsal sides and Figure S6 for the ventral sides). Associated colour patterns correspond to lighter, striped colour patterns vs. darker, spotted colour patterns, respectively. Such strong associations between wing shape and colour patterns remaining when controlling for the effect of phylogenetic relatedness between species suggest that some selective pressures might have favoured certain trait combinations.

We did not find any significant effect of species main biome or host‐plant on the tail shape/colour pattern associations (Tables S3 and S4), preventing us from drawing any conclusions on the effect of micro‐habitats on the evolution of specific trait combinations.

### 3.2 Tail Elongation Is Associated With Marginal Spots and Contrasted Markings

**FIGURE 3:**
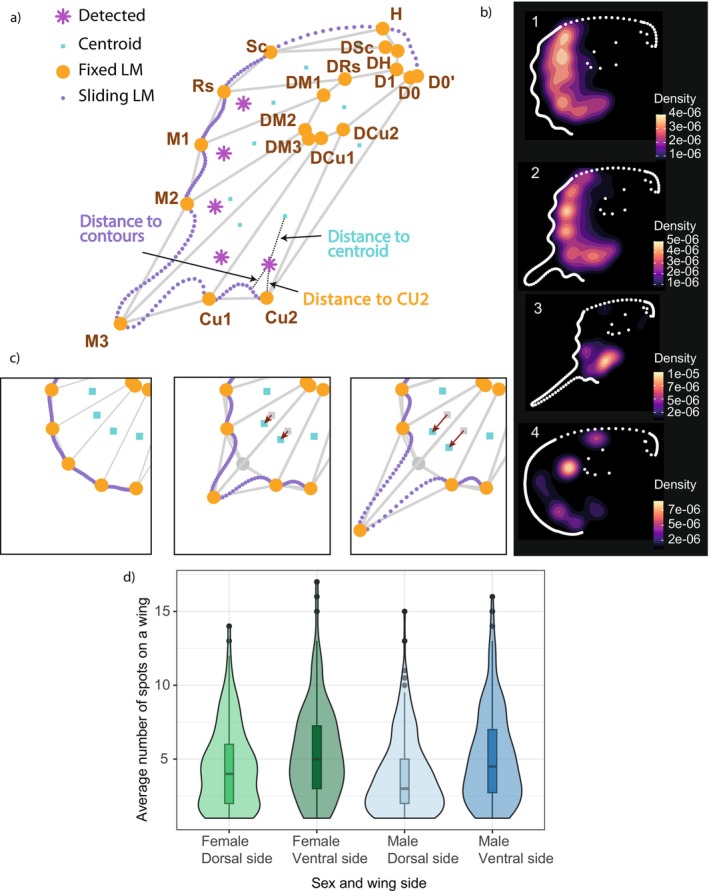
Detection and measurement of spots on the hindwings. (a) Schematic positions of the landmarks used to describe wing shape, and of the measurements performed to characterise the localisation of the spots (example taken from the ventral side of *Pachliopta liris*). (b) Density of localisation of all detected dorsal spots on the mean wing shapes for each of the four clusters of male wing shape. (c) Illustration of the shift of wing cell centroids with tail elongation. From left to right panel, the landmark M3 was incrementally artificially displaced towards the bottom left to elongate the tail, and the centroids were recalculated. The red arrows show centroid displacement from the original wing. (d) Distribution of the average number of spots on hindwings between sexes and wing side shows a higher number of spots on ventral than dorsal side (Females dorsal side‐Females ventral side: *p =* 0.0003, Males dorsal side‐Males ventral side *p* = 4.2e‐05).

We then investigated the evolution of specific colour pattern features that may impact the visual effect in species displaying different types of hindwing tails, using PGLS analyses. We first tested for the correlated evolution of the position of conspicuous coloured spots on the wings with tail elongation (Table 1). Under the hypothesis that tails play a role in predation deflection alongside contrasting spots, longer tails may be associated with spots located near the wing margins, meaning that the minimum distance from the spot to the wing contours would be smaller and/or near the tail base (CU2 landmark, see Figure 3).

More spots were detected on the ventral wing surface in both sexes (Figure 3d). When considering both tailed and tailless species, we found that tail elongation is associated with two types of spot positions. First, species with longer tails show a strong association with spots located closer to the wing contours and to the base of the tail. Second, a positive interaction between distance to centroids and distance to contours (FV, *p* < 0.05, MV, *p* < 0.05) indicates that, in some species, longer tails are also associated with spots that deviate from their developmentally expected position (high distance to centroids), but only when these spots are positioned far from the wing contours (high distance to contours).

When restricting our dataset to tailed species (*n* = 114 species for females and *n* = 149 species for males), we found that thin, long tails are only associated with spots closer to the CU2 landmarks and thus closer to the tail base.

Secondly, we tested for the correlated evolution of broad colour pattern characteristics with tail elongation, using granularity analyses within the ImageJ MICA Toolbox. Particularly, we tested for correlation of tail elongation with marking size, contrast, and marking dominance, that is, the prominence of the most contrasted markings compared to other markings on the wings, which is a measure of pattern complexity (Table 2).

In males, tail elongation was associated with finer and more contrasted markings (such as the fine contrasted stripes of *Protographium molops*, Figure 2C). Marking size also had interactions both with marking contrast and marking dominance on the ventral side of males: large, contrasted markings (MV, *p* < 0.01) and a more complex colour pattern (MV, *p* < 0.01) tend to be associated with tail elongation as well (see *Papilio alexanor* with mixed spots and broad stripes, Figure 2C). Surprisingly, we did not find any association between colour pattern descriptors and female tail elongation except finer markings associated with tail elongation in the dorsal side of females (FD, *p* < 0.05).

When restricting our dataset to tailed species, we found similar associations between colour pattern descriptors from the granularity analysis and small spatulated tails vs. thin and long tails. For both sexes, we found that spatulated tails are marginally associated with larger markings, but that this association is significant for colour patterns that are less complex (see for example *Parides bunichus* that display only one type of red spots, Figure 2C), and that larger and more contrasted markings tend to be associated with thinner and longer tails.

## 4 Discussion

### 4.1 Preferential Associations of Tail Shape and Colour Pattern

This study reveals a significant correlation between colour pattern variation and tail shape variation across species, even after accounting for shared evolutionary history. We first assessed the correlation between the whole colour pattern and tail shape and then focused on particular features of the colour patterns. Whole colour pattern assessment unveiled associations from dark, spotted patterns with short and spatulated tails to lighter, striped patterns associated with tail elongation. Species displaying shorter tails and spotted patterns included unpalatable species, such as *Pachliopta hector*, or species belonging to mimicry rings, such as *Papilio erostratus* (Figure 2). Linke et al. (2022) have shown that wing shape and particularly hindwing tails of butterflies can be used as visual cues by predators to identify aposematic species. As unpalatable species may retain toxic compounds from their host‐plant, we tested for an effect of host‐plant on tail shape/colour pattern association but the effect of host‐plant used on this association became non‐significant after accounting for phylogeny, preventing robust conclusions on the putative effect of selection linked to the consumption of host‐plant with noxious compounds. Host‐plants tend to be conserved among clades (Allio et al. 2021), and the close resemblance of models and mimics that do not share a similar host‐plant may partly explain this lack of effect. Moreover, the lack of large‐scale experimental evidence for unpalatability in the short‐tailed species prevents any robust conclusions on the aposematism associated with these colour patterns.

Conversely, across Papilionidae species, wings with longer, thinner tails were significantly more associated with striped patterns. These associations hold when accounting for phylogeny, meaning that similar associations between colour pattern and tail shape can be found in distantly related species. For instance, the distantly related species *Iphiclides podalirius* and *Papilio alexanor* (Figure 2) display striped patterns associated with thinner tails, pointing at a possible convergent effect of natural selection favouring such association of visual features. These types of contrasted, striped markings may be involved in interactions with predators in several ways. First, large contrasting longitudinal stripes may disrupt the outlines of the butterfly wings, making the shape of the wings less recognisable and less detectable by the predators (Stevens 2007). Secondly, repeated high contrast stripes are likely conspicuous to predators, but may function as dazzle markings. Indeed, highly contrasted lines or bands in moving prey have been shown to impact predator attention, leading to erroneous estimation of prey speed and reduced attack success (Kodandaramaiah et al. 2020). Even in prey with a stationary behaviour, the crowding effect of the repeated lines on the neuronal response of predators could lead to predator confusion and reduce attack success (Stevens 2007). Several studies on various taxa have shown an effect of the combination of stripes, body elongation and particular movements on predator attacks, deflecting strikes to posterior, non‐vital body parts (Jackson et al. 1976; Murali and Kodandaramaiah 2016; Urban et al. 2022). The frequent association of striped pattern and long tails in *Papilionidae* could thus stem from a synergistic effect of both traits on predator deflection. In particular, the combination of stripes acting as dazzle markings (Murali and Kodandaramaiah 2016) and marginal contrasting spots that redirect predator attention towards the rear of the butterflies may provide a survival advantage. This advantage could be reinforced by the long tail appendages that may break off more easily than the rest of the wings (Chotard et al. 2022) but also attract predator attention. The combination of such colour pattern and hindwing tail could thus be co‐selected under predation pressure. Alternatively, highly contrasted patterning can also be involved in aposematism or other kinds of signalling (such as crypsis/aposematism, for example; Barnett et al. 2017). The lack of effect of either the biome structure or sexual selection on the tail shape/colour pattern associations (Data S1: Section 4) further supports a key role of predator‐mediated interactions in the co‐evolution between hindwing tail and colour pattern.

Species may display variations along the continuum of tail shape and colour pattern association, such as many species of *Papilio* that are neither aposematic nor mimetic and display tails associated with broad band patterns and marginal spots on the hindwings. We nevertheless detected preferential associations consistent with the hypothesis of two broad categories of visual features, as supported by a clustering analysis on PLS coordinates (Data S1: Section 7), potentially reflecting warning or deflective effects (see Figure 2c for an illustration). Further experimental work is now needed to investigate the selective pressures underlying these main tail shape/colour pattern associations.

### 4.2 Specific Colour Pattern Features Are Associated With Tail Elongation

We identified the key features driving tail shape/colour pattern associations: we found that tail elongation is associated with contrasting spots near the wing outline and CU2 landmark, as well as finer, more contrasting markings. Here, we studied all contrasting spots on the hindwings, as contrast is a major component of visual signal affecting predator attention (Stevens et al. 2008, 2009). Although we did not distinguish eyespots from simple contrasting spots, eyespots have been implicated in sexual signalling (Breuker and Brakefield 2002; Huq et al. 2019; Robertson and Monteiro 2005) and predation deflection (Iserhard et al. 2024; Lyytinen et al. 2004; Olofsson et al. 2010). However, when we assessed the association between tail shape and spots containing multiple colours (that may be closer to the classical definition of eyespots, Monteiro et al. 2006), we found similar trends than when considering all spots (Data S1: Section 5). Additionally, predation experiments on *Iphiclides podalirius* showed preferential attacks on a combination of tails and conspicuous marginal spots, suggesting their implication in deflecting attacks (Chotard et al. 2022). The association of contrasting markings and marginal spots with longer tails suggests joint selection pressure on tail shape and colour pattern due to predation. Conversely, shorter tails are associated with larger and less complex markings, meaning that one type of marking dominates the colour pattern. Larger markings and simpler colour patterns may enhance predator avoidance behaviour by increasing detectability and recognition of aposematic colour patterns (Forsman and Merilaita 1999; Stevens and Ruxton 2011). These consistent associations between colour pattern features and hindwing tail shape suggest morphological patterns potentially reflecting predation‐mediated selection.

### 4.3 Joint Evolutionary History of Tail Shape and Colour Pattern Diversification

By independently estimating the rates of evolution of hindwing tail shape and of whole colour pattern, we found positive correlations ranging from 0.27 to 0.41 throughout the phylogenetic tree. Estimating phenotypic rates across a phylogenetic tree strongly depends on the branch length distribution (Castiglione et al. 2018): longer branches tend to result in slower rates of evolution. This dependence may bias the estimation of the rate correlations between phenotypic traits, and such estimates may be artificially inflated and should be interpreted with caution. Nevertheless, we found that increased diversification of tail shape was concomitant with increased diversification of colour pattern. The sequence of this joint evolution remains unknown, whether the apparition of tails preceded subsequent changes in colour patterns or *vice‐versa*. Longer tails could evolve under selective pressure stemming from aerodynamic performances (Park et al. 2010) and then be co‐opted as a part of the visual signal with aposematic or deflective colour patterns. Conversely, deflective colour patterns may evolve first, as marginal spots can have a deflective effect without the presence of a tail (Olofsson et al. 2010) and then drive the evolution of longer tails that enhance the deflective effect. Correlated diversification supports joint evolution under shared selection pressures or genetic/developmental constraints (Felice et al. 2018; Klingenberg 2008). Genetic associations between wing colour pattern and shape are well‐documented in mimetic butterflies, where supergenes or single‐locus architectures control both tail presence and colour pattern variation (Komata et al. 2016; Palmer and Kronforst 2020; Timmermans et al. 2014; Yoda et al. 2021). Correlated selection on traits is likely to generate subsequent genetic associations between traits (Svensson et al. 2021), as is the case in mimetic syndromes, so that the genetic architecture controlling colour and shape variations could itself also be shaped by the specific selective regime involved.

Developmental correlations between coloured spots localisation and wing shape and venation have also been specifically studied. Wing shape variation often implies wing cell shape variation: elongated wings tend to have elongated wing cells as well, likely impacting the position of the spots (Debat et al. 2020). Indeed, hindwing tails and scalloped wing margins are often accompanied by dislocation of neighbouring spots (McKenna et al. 2020). Moreover, in *Bicyclus anynana* butterflies, artificial selection showed that wing cell shape changes are accompanied by eyespot shape changes (Monteiro et al. 1997). Here, we assumed that spots should stand at the centroid of each wing cell, as a result of developmental constraints, as summarised in the Papilionidae developmental ground plan (Mazo‐Vargas et al. 2024). We tested whether these developmental expectations were met by measuring the deviation between the actual spot localisations and the centroids and found no general departure, and thus spots may be closer to the wing outline simply because of developmental constraints. However, we did find a significant deviation from the developmental expectation in species with spots further from the wing outlines on the ventral sides. This suggests that the evolution of spots that are far from the wing outlines in tailed species may be influenced by selective pressures (e.g., the aposematic species *Parides tros* and *Losaria neptunus*), driving the spots away from their developmentally expected location. Alternatively, the hypothetical groundplan centroids assumed here might be too conservative, and the development of elongated wing cells does not imply a strictly allometric displacement of morphogen foci of the same magnitude. This interpretation, however, depends on the precision of this developmental prediction that would require specific analysis of the expression of patterning genes.

Interestingly, we also found an association between longer tails and a preferential position of spots near the CU2 landmark, as illustrated in Figure 3b. These spots, located close to the anal edge, may deflect attacks towards the tail base (Chotard et al. 2022). The regionalization of colour pattern regulatory genes expression on the wings (McKenna et al. 2020) may allow for the expression of contrasting spots preferentially in the expression domains near the tail base, compared to the rest of the wing. Preferential expression of conspicuous CU2‐adjacent spots in long‐tailed species could thus be promoted by natural selection by predators. While wing shape and colour pattern are developmentally linked by the wing venation pattern, selective pressures could promote the expression of region‐specific pattern elements, such as these contrasted spots near the tail base and tail elongation.

## 5 Conclusions

We uncover evidence for correlated evolution of colour pattern features and tail shape variation in wings of swallowtail butterflies, particularly between specific colour pattern features associated with predation deflection and tail shape elongation. Although both of these complex traits are likely to be influenced by multiple selective pressures, our results suggest that natural selection by predators leads to their co‐selection on a macroevolutionary scale, resulting in the emergence of predation‐driven morphological syndromes.

## Author Contributions

A.P. performed the machine learning and computer vision analyses and the phylogenetic analyses. A.C. collected the photographic data and the geometric morphometric landmarks data. All authors designed the research and wrote the paper.

## Conflicts of Interest

The authors declare no conflicts of interest.

## Supporting information


**Data S1:** ele70303‐sup‐0001‐Supinfo01.docx.

## Data Availability

R and Python codes as well as colour pattern and tail shape data are available at https://github.com/AgathePuissant/Correlation_tail_colour_evolution.
