# Supplementary material for: The Coevolution of Colour Patterns and Hindwing Shapes on a Large Phylogenetic Scale Reveals Predation‐Driven Adaptive Syndromes in Swallowtail Butterflies
Source: Ecol Lett. 2026 Jan 18;29(1):e70303. doi: 10.1111/ele.70303 (PMC12812247; doi:10.1111/ele.70303)
Supplement: Supplementary file 1 — Data S1: ele70303‐sup‐0001‐Supinfo01.docx. [file ELE-29-0-s001.docx]

# **Supplementary Information**

1. Method details
   1. SimCLR training

SimCLR training involves modifying the original images (cropping, rotations…) so the network learns to recognize discriminative features of the different images: the network is indeed trained to distinguish modified versions of the same image (considered to be similar) from modified versions of different images (considered to be dissimilar). After training, the unmodified original images are submitted to the neural network, generating 2048-dimensional embedding vectors capturing discriminative image features.

We chose the image modifications to bias the learning toward image features that do not depend on wing shape. We thus performed image rotations, image flipping, and image cropping up to 20% of the original image size. These specific modifications used during training prevent the neural network to use wing shape features as the discriminant features between images. The quantification of similarity between images then mostly depends on variations in colour pattern features rather than on variations in shape (see Puissant et al., 2023 for more details). Nevertheless, to remove any spurious correlation between our colour pattern machine learning embeddings and geometric morphometrics data, we removed the tails in our specimen images using custom Python code with the *opencv* package and used these images to train SimCLR and retrieve colour pattern embeddings.

##

## Geometric morphometrics

The anal outline was excluded as this part of the wing is often folded, missing or shows inner androconial brushes on some species. Landmarks and semi-landmarks were digitized using TpsDig2 (Rohlf, 2015) and were then superimposed using a generalized Procrustes analysis (Rohlf & Slice, 1990) implemented in the *gpagen* function of the R package *geomorph* (Adams & Otárola-Castillo, 2013). The semi-landmarks were slided by minimising the bending energy (Mitteroecker & Gunz, 2009).

## Ecological data acquisition

We retrieved the main host plants for each species from Allio *et. al.* (2021). We retrieved each species main biome using their geographical ranges shapefiles, taken from Puissant *et. al.* (2023) which comprises 125 ranges exported from the Map of Life project (Jetz et al., 2012) or from the IUCN Red List Data & Mapping resources and 101 ranges generated from cleaned and filtered GBIF data. 108 additional ranges were generated using a literature review on species range (*e.g.* Nakae et al., 2021; Scriber et al., 1995; Tyler et al., 1994) and the *sf* package in *R*. Then, we used the RESOLVE Ecoregions 2017 dataset (Dinerstein et al., 2017) to get the biome covering the largest percentage of area on each species range. The Ecoregions dataset delimitates global land cover into 846 ecoregions, classified into 14 different biomes: (1) Tropical and subtropical moist broadleaf forests, (2) Tropical and subtropical dry broadleaf forests, (3) Tropical and subtropical coniferous forests, (4) Temperate broadleaf and mixed forests, (5) Temperate conifer forests, (6) Boreal forests or taiga, (7) Mangroves, (8) Tropical and subtropical grasslands, savannas, and shrublands, (9) Temperate grasslands, savannas, and shrublands, (10) Flooded Grasslands and Savannas, (11) Montane grasslands and shrublands, (12) Tundra, (13) Mediterranean forests, woodlands, and scrub, (14) Deserts and xeric shrublands.

## Granularity analysis

This technique describes colour pattern elements by analyzing the colour pattern at various spatial scales. The image is filtered into different layers, each corresponding to a specific range of spatial frequencies (from 2 pixels to 512 pixels with a multiplicative increment of $\sqrt{2}$, broadly corresponding to spatial frequencies smaller than the wing sizes). The energy of a signal at a given filter size is calculated as the standard deviation of the pixel values, for the pixels associated with this signal.

Higher magnitude (or energy) at a particular frequency is associated with repeated elements of this particular marking size. For each image, we obtain an energy spectrum describing the energy associated with every filter size. From this energy spectrum, we extract three quantities: (1) the maximum energy, corresponding to the presence of more contrasting dominant elements in the colour pattern, (2) the frequency of the maximum energy peak, corresponding to the size and spacing of the dominant markings, divided by wing area to get relative marking size, and (3) the proportion of maximum energy to the sum of energies, corresponding to how much a particular pattern dominates the overall colour pattern, which is a measure of colour pattern complexity.

## Detection of spots on the hindwings

We separated the hindwings with the tails removed for each specimen and discretized colours on the images using the R package *recolourize* (Weller et al., 2024) with a fixed number of colours for each wing. For each different colour we obtained a binary mask. We then applied morphological operations such as smoothing and noise removing on this mask. Then, we used contour detection to detect shapes that tended to be circular using tailored parameters with the *opencv* Python package.


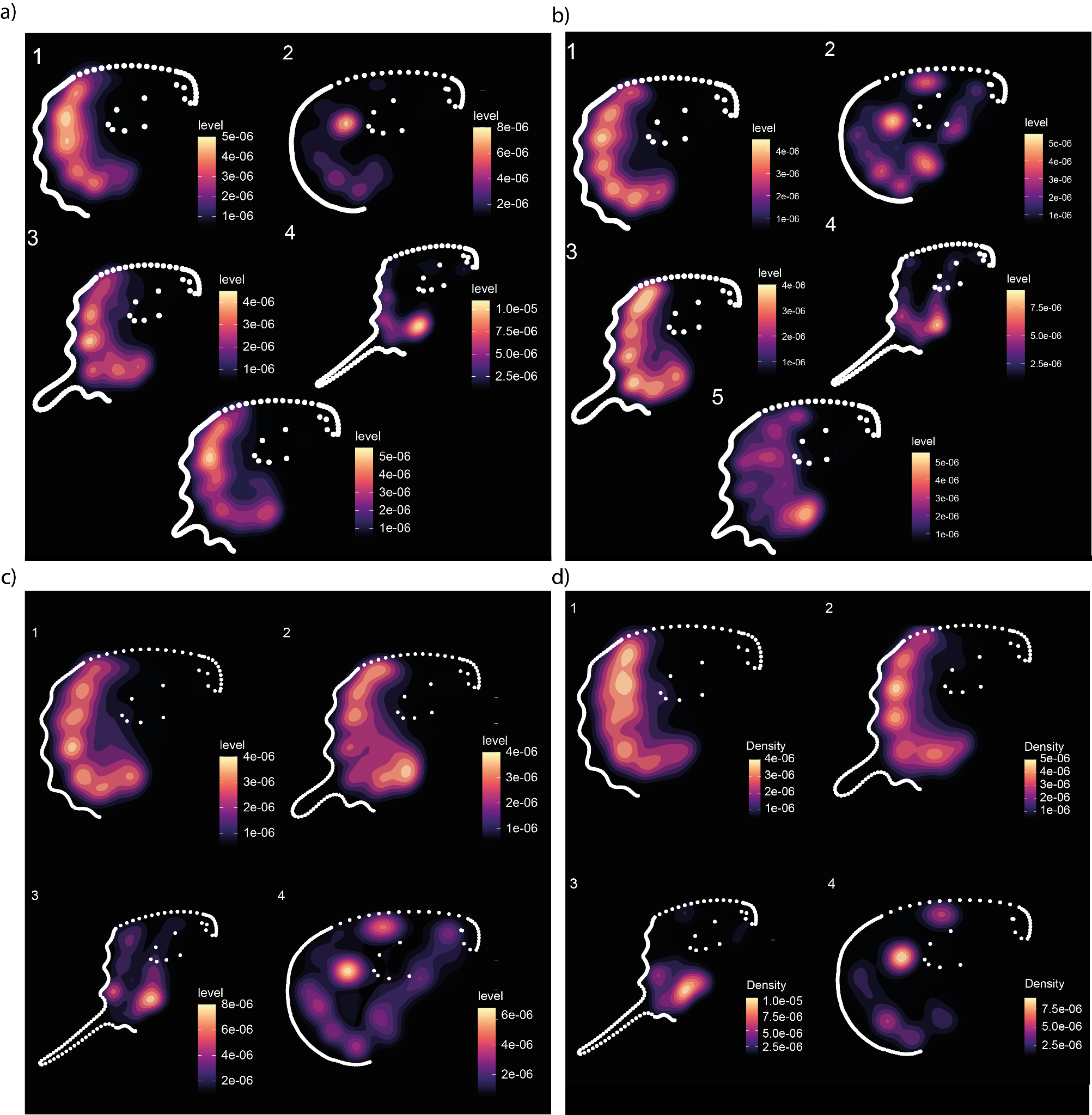


Figure 1. Density of localisation of detected spots on the mean wing shapes for the five clusters of female wing shape and for the four clusters of male wing shape. a) Female, dorsal sides. b) Female, ventral sides. c) Male, dorsal sides. d) Male, ventral sides.

## Correlated rates of evolution


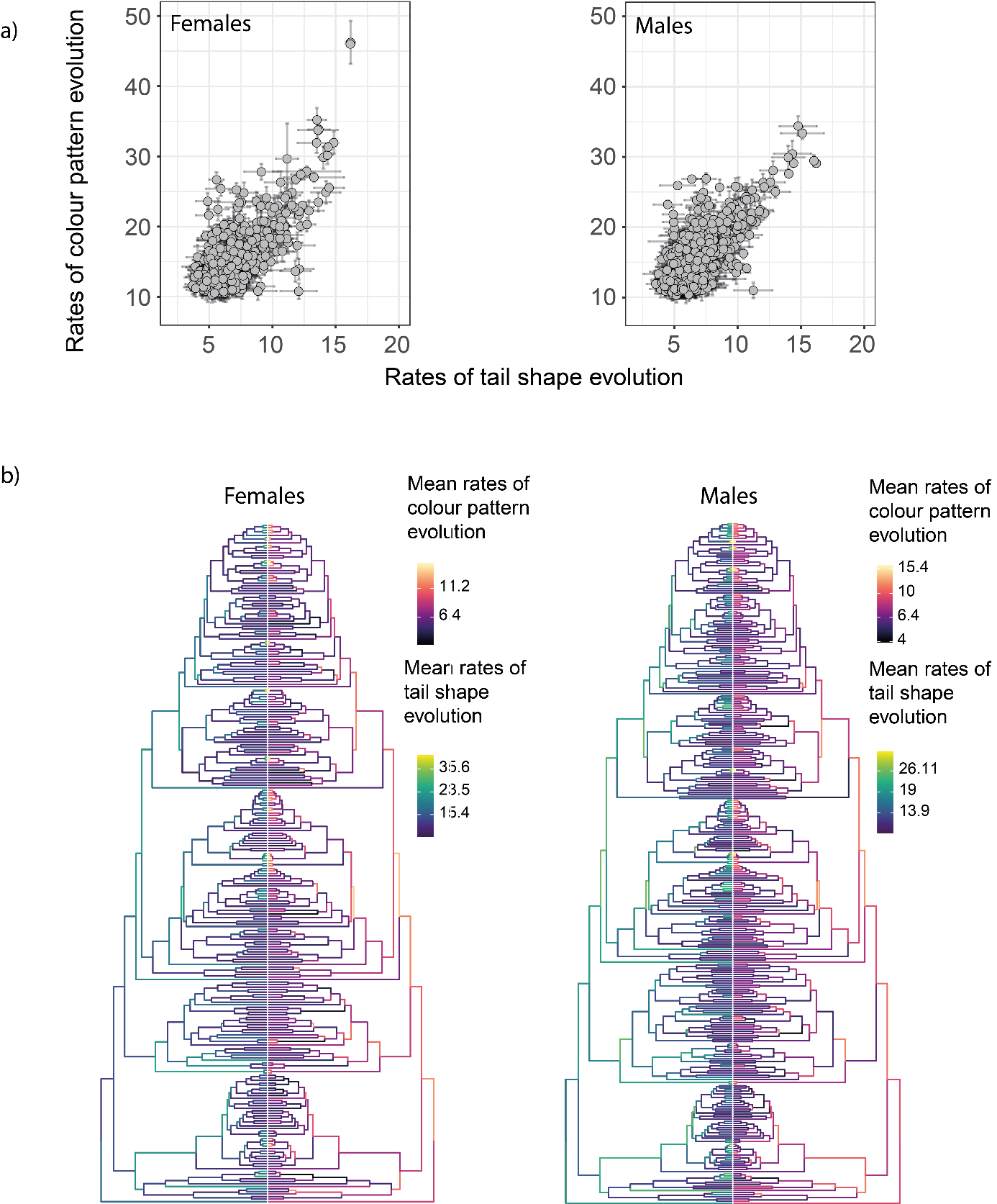


Figure 2. Correlated multivariate rates of tail shape and ventral colour pattern evolution along the phylogeny. a) Distribution of R² between tail shape and ventral colour pattern rates for the 100 intraspecific sampling for females and males. b) Mean branch rates of ventral colour pattern evolution *vs.* mean branch rates of tail shape evolution with the standard deviation of the 100 intraspecific sampling as error bars for females, and c) for males. d) Mean branch rates of tail shape (left) and ventral colour pattern (right) for the 100 intraspecific sampling mapped onto the phylogeny for females, and e) for males.

## Two block PLS and phylogenetic two block PLS

We calculated the tail length as the distance between the M3 landmark and the midpoint between the M1 and CU2 landmark.


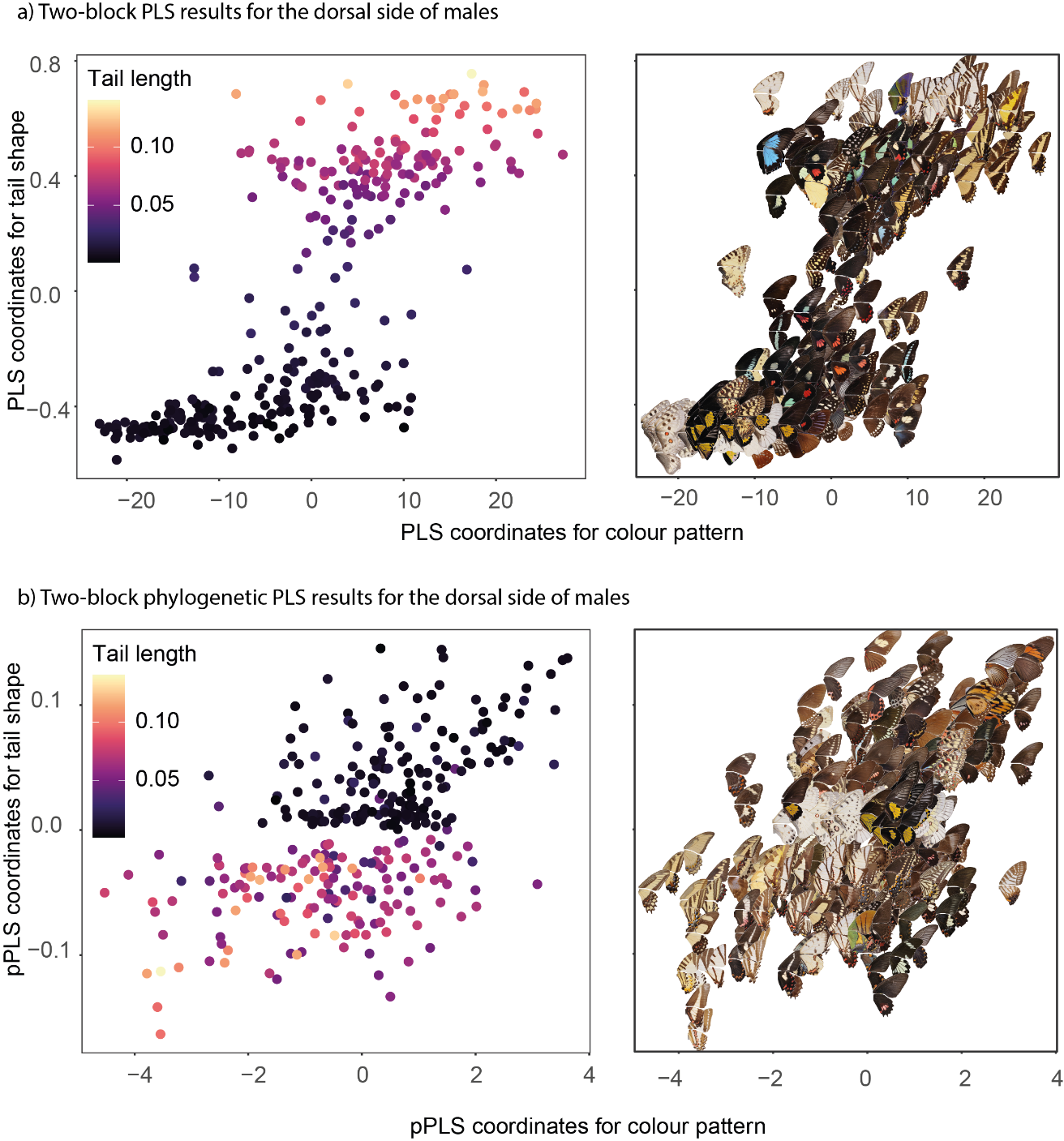


Figure 3. 2B-PLS and phylogenetic 2B-PLS show associations driven by hindwing tail presence/absence. a) Projection of tail shape and colour pattern on the first block of covariation for the 2B-PLS found for the dorsal side of males. Left: Projection with tail length mapped on each species. Right: Projection with example pictures of phenotypes with tail removed mapped on each species. b) Projection of tail shape and colour pattern on the first block of covariation for the phylogenetic 2B-PLS found for the dorsal side of males. Left: Projection with tail length mapped on each species. Right: Projection with example pictures of phenotypes with tail removed mapped on each species.

Two-block PLS results

|  |  | Females | Males |
| --- | --- | --- | --- |
| R-PLS for all species | Dorsal | 0.719 (P<0.001) | 0.763 (P<0.001) |
|  | Ventral | 0.75 (P<0.001) | 0.795 (P<0.001) |
| R-PLS for tailed species | Dorsal | 0.624 (P<0.001) | 0.695 (P<0.001) |
|  | Ventral | 0.569 (P<0.001) | 0.636 (P<0.001) |

Table 1. R-PLS obtained from 2B-PLS between multivariate tail shape data and multivariate colour pattern coordinates.

Two-block pPLS results

|  |  | Females | Males |
| --- | --- | --- | --- |
| pR-PLS for all species | Dorsal | 0.51 (P<0.05 for all 1000 samplings) | 0.54 (P<0.05 for all 1000 samplings) |
|  | Ventral | 0.56 (P<0.05 for all 1000 samplings) | 0.62 (P<0.05 for all 1000 samplings) |
| pR-PLS for tailed species | Dorsal | 0.48 (P<0.05 for all 1000 samplings) | 0.50 (P<0.05 for all 1000 samplings) |
|  | Ventral | 0.50 (P<0.05 for all 1000 samplings) | 0.50 (P<0.05 for all 1000 samplings) |

Table 2. Mean pR-PLS across 1000 intraspecific re-samplings, obtained from phylogenetic 2B-PLS between multivariate tail shape data and multivariate colour pattern coordinates.


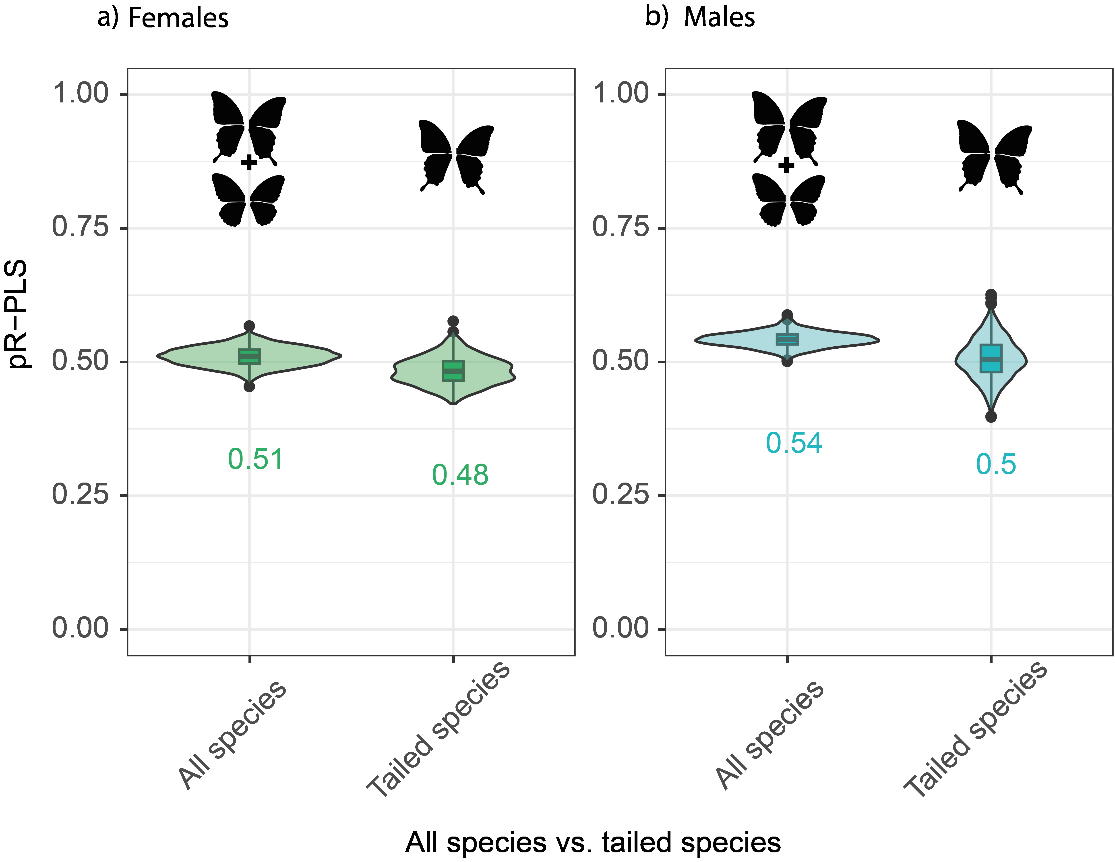


Figure 4. Significant associations between tail shape and dorsal colour pattern after correcting for phylogenetic distances between species. a) pR-PLS distribution for the 1,000 analyses performed on the intraspecific re-sampling among all species (left) and among tailed species (right) for females, and b) for males. Median value of the distribution is displayed below the violin plots. Among the 1,000 analyses performed on the intraspecific re-sampling, significant phylogenetic two-block PLS was consistently observed (100% of the tests provided a *P*<0.05).


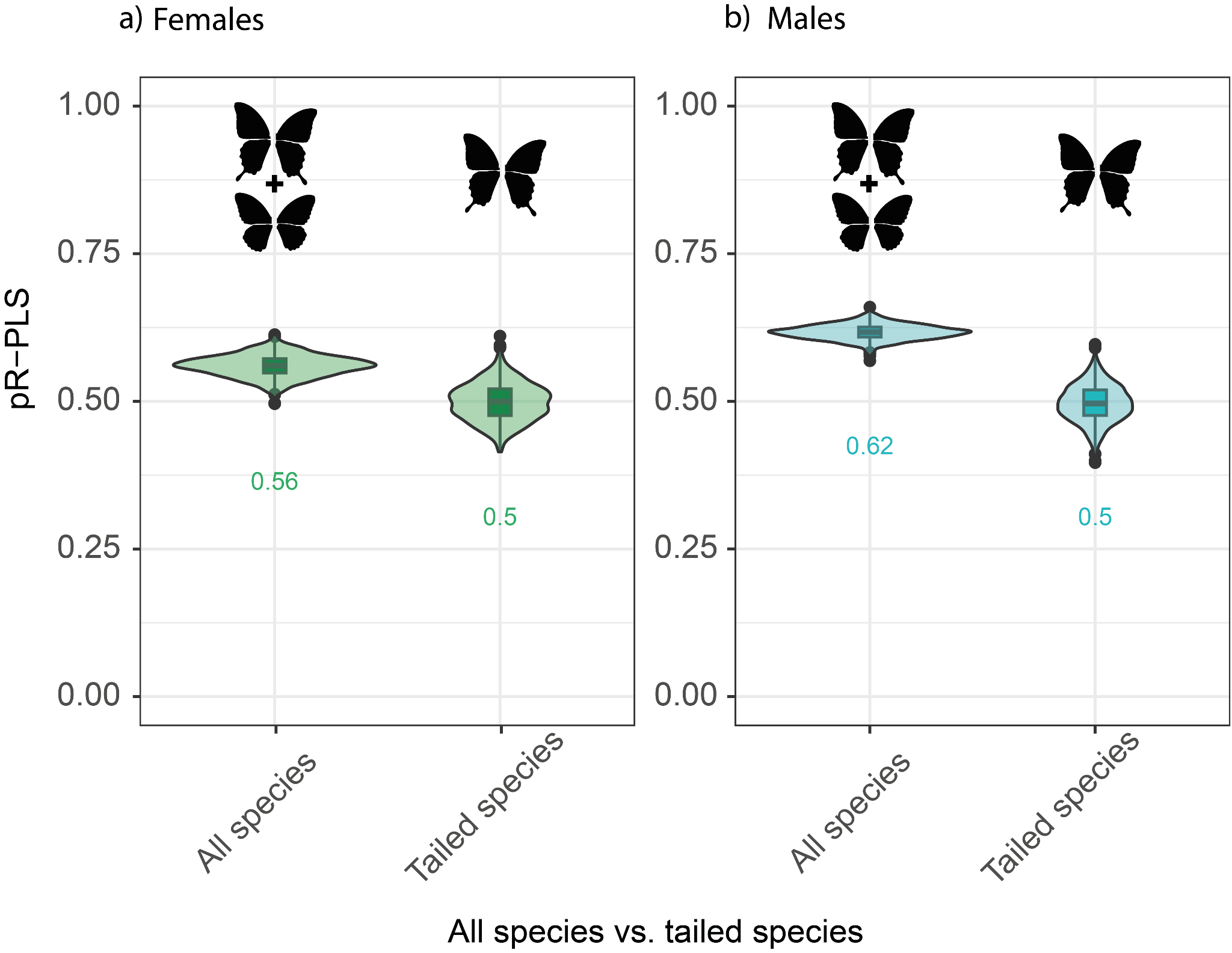


Figure 5. Significant associations between tail shape and ventral colour pattern after correcting for phylogenetic distances between species. a) pR-PLS distribution for the 1000 analyses performed on the intra-specific re-sampling among all species (left) and among tailed species (right) for females and b) for males. Among the 1000 analyses performed on the intra-specific re-sampling, significant phylogenetic two-block PLS was consistently observed (100% of the tests provided a *P*<0.05).


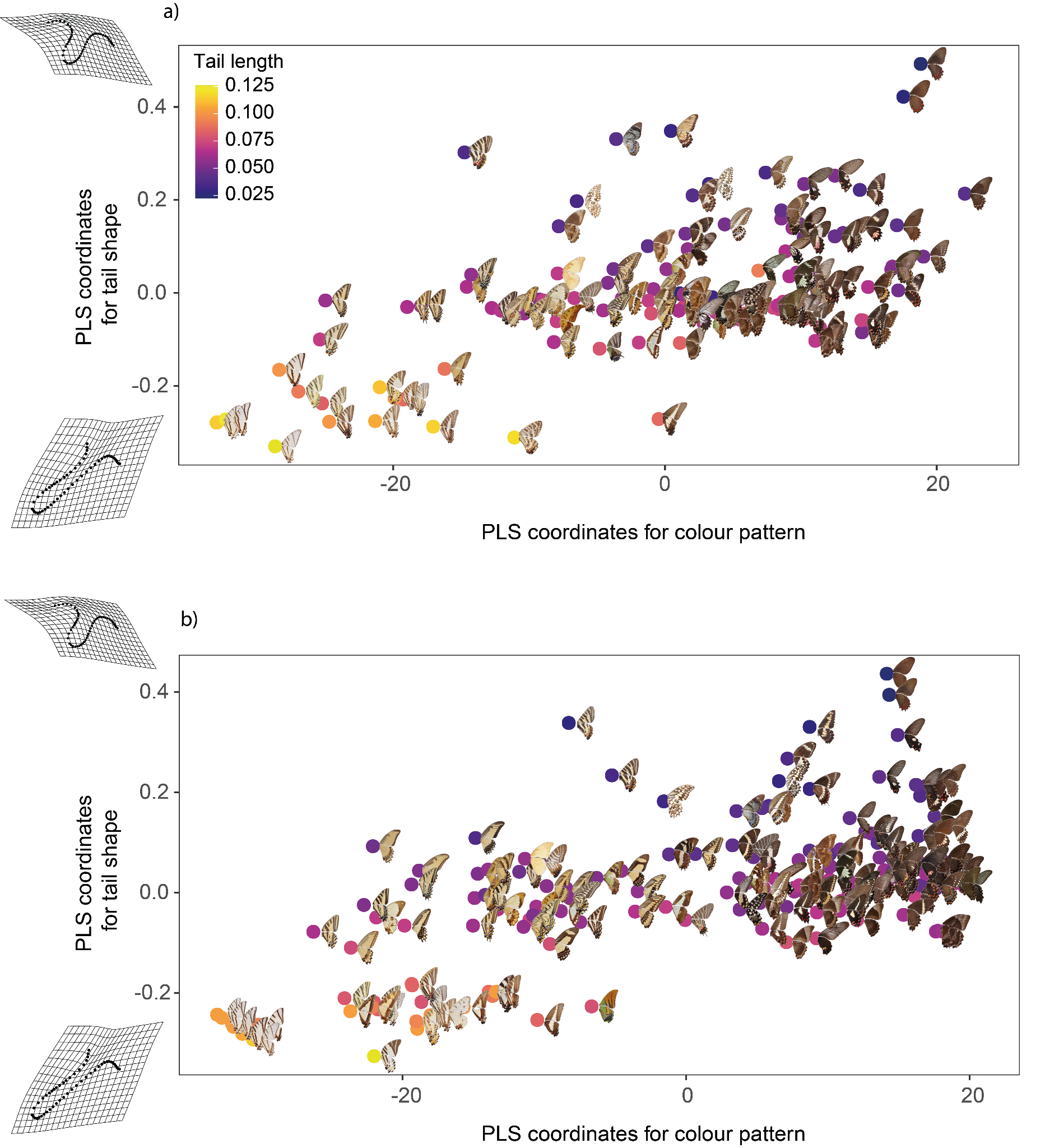


Figure 6. Significant associations detected between tail shape and tail-removed ventral wing colour pattern, showing that longer tails are associated with striped patterns. a) Two-block PLS projection between tail shape and colour pattern for females (pR-PLS = 0.5, *P*<0.05), b) Two-block PLS projection between tail shape and colour pattern for males (pR-PLS = 0. 5, *P*<0.001).

|  | *All species* | *Tailed species only* |
| --- | --- | --- |
| *Females (dorsal)* | Pillai: 0.0957, p = 0.3656 (NS) | Pillai: 0.3008, p = 0.2208 (NS) |
| *Females (ventral)* | Pillai: 0.0901, p = 0.4236 (NS) | Pillai: 0.2666, p = 0.3327 (NS) |
| *males (dorsal)* | Pillai: 0.0619, p = 0.5754 (NS) | Pillai: 0.1466, p = 0.1409 (NS) |
| *Males (ventral)* | Pillai: 0.0502, p = 0.6823 (NS) | Pillai: 0.2214, p = 0.2987 (NS) |

Table 3. Results from multivariate phylogenetic regression of species main host-plant on 2B-PLS coordinates.

|  | *All species* | *Tailed species only* |
| --- | --- | --- |
| *Females (dorsal)* | Pillai: 0.1102, p = 0.1139 (NS) | Pillai: 0.3439, p = 0.3207 (NS) |
| *Females (ventral)* | Pillai: 0.1070, p = 0.1329 (NS) | Pillai: 0.3711, p = 0.2438 (NS) |
| *Males (dorsal)* | Pillai: 0.0755, p = 0.2188 (NS) | Pillai: 0.1210, p = 0.4496 (NS) |
| *Males (ventral)* | Pillai: 0.0738, p = 0.2378 (NS) | Pillai: 0.0301, p = 0.3087 (NS) |

Table 4. Results from multivariate phylogenetic regression of species main biome on 2B-PLS coordinates.

## Sexual dimorphism of tail shape and colour pattern

We calculated colour pattern dimorphism for each phenotypic form by taking the Euclidean distance between colour pattern coordinates of males and females, and the tail shape dimorphism by taking the Euclidean distance between tail shape coordinates of males and females.

## Little evidence for influence of environmental natural selection or sexual selection on the correlated evolution of tail shape and colour pattern

The evolution of tail shape and colour pattern may independently be subjected to selection by abiotic factors linked to local biome, such as ambient light conditions or habitat structure (Chazot et al., 2016, 2021; Dalrymple et al., 2018; Le Roy et al., 2019). We tested for an effect of biome on the association of tail shape and colour pattern, using coarse biome classification, and found no significant effect after accounting for phylogenetic relationships. This suggests that environmental factors are probably not a main driver of the joint evolution of tail shape and colour pattern. However, additional data on species microhabitat are needed to test more specific hypotheses, such as the influence of flying in open or closed microhabitat on tail elongation and colour pattern evolution.

Sexual selection for the complexity of ornaments could also theoretically drive the evolution of contrasting and conspicuous colour patterns combined with tail elongation (Cui et al., 2016; Miles & Fuxjager, 2018; van der Bijl et al., 2020). We computed sexual dimorphism in each species, and found correlated dimorphism of tail shape and colour pattern, but species that were highly dimorphic in both traits corresponded to polymorphic species involved in female-limited mimicry, such as *Papilio memnon* and *Papilio dardanus* (Supp. Mat. Figs. 7 and 8). In Papilionidae, colour pattern dimorphism at a macroevolutionary scale is mostly driven by female divergence towards mimetic forms more than male divergence (Kunte, 2008; Puissant et al., 2023). Moreover, geometric morphometric studies in Papilio have found no tail shape dimorphism in non-mimetic species (Koutrouditsou & Nudds, 2021; Owens et al., 2020), and drastic tail shape variation has been linked to Batesian mimicry (Owens et al., 2020). Additionally, mate choice experiments on *Actias luna* moth, which likely rely on visual cues for mating, have shown no effect of tail presence/absence on reproductive success (Rubin & Kawahara, 2023). Sexual selection may impact colour pattern evolution in Papilionidae, but evidence for an effect of sexual selection on tail shape evolution remains limited. Given that the correlated evolution of both traits is found to be minimally influenced by sexual selection, although the evolution of dimorphism may be partially driven by sexual selection, the hypothesis that predator-mediated interactions play an important role in this co-evolution remains the most likely explanation.


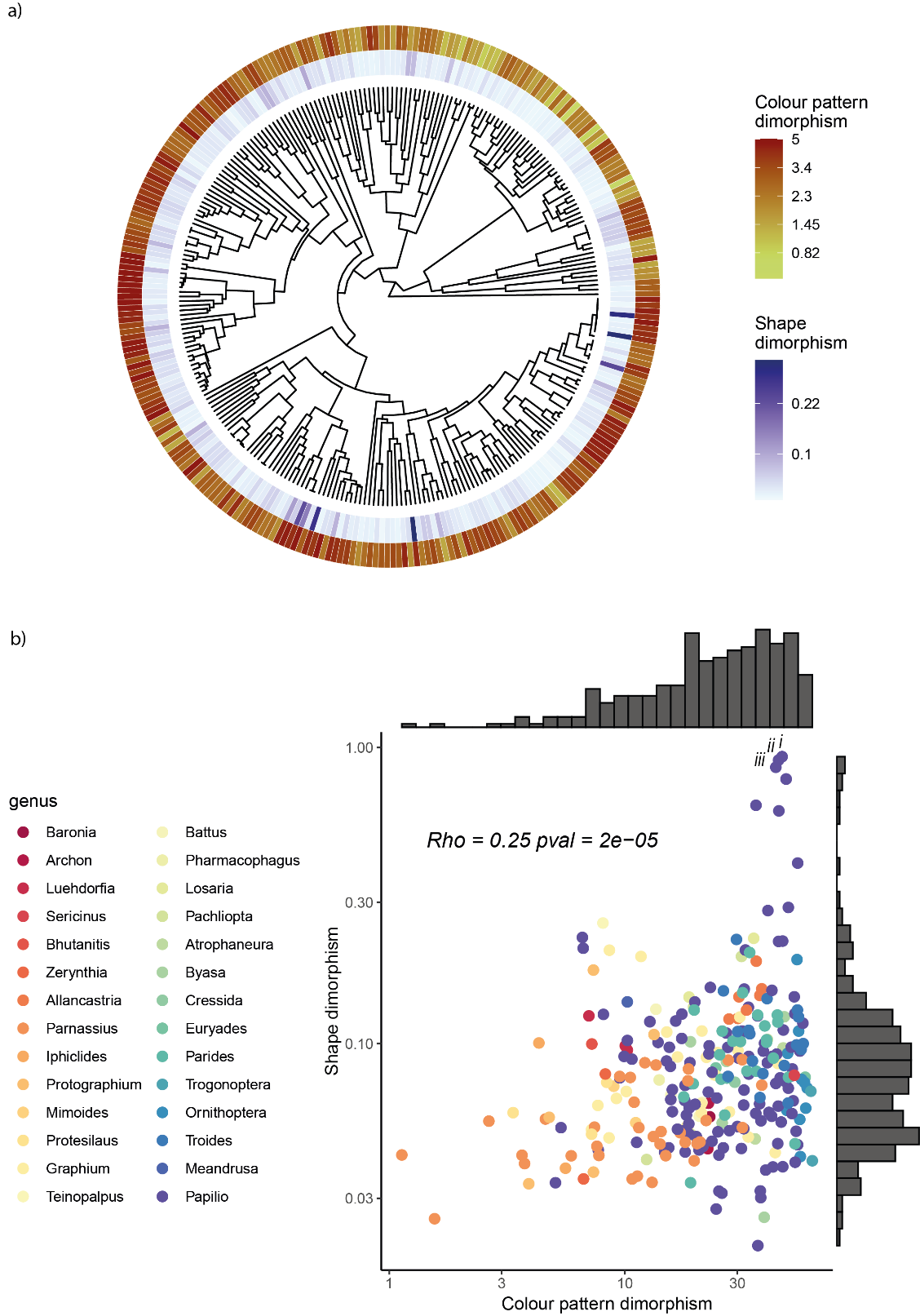


Figure 7. Correlation of shape and dorsal colour pattern dimorphism at the macroevolutionary scale. a) Shape and dorsal colour pattern dimorphism along the phylogeny. b) Distribution of shape and dorsal colour pattern dimorphism and rank correlation (Spearman rho = 0.25, *P*<0.001). The colours correspond to the genus, the x and y axis are log scaled. i: *Papilio dardanus*, ii: *Papilio memnon achates*, iii: *Papilio memnon imperiosa.*


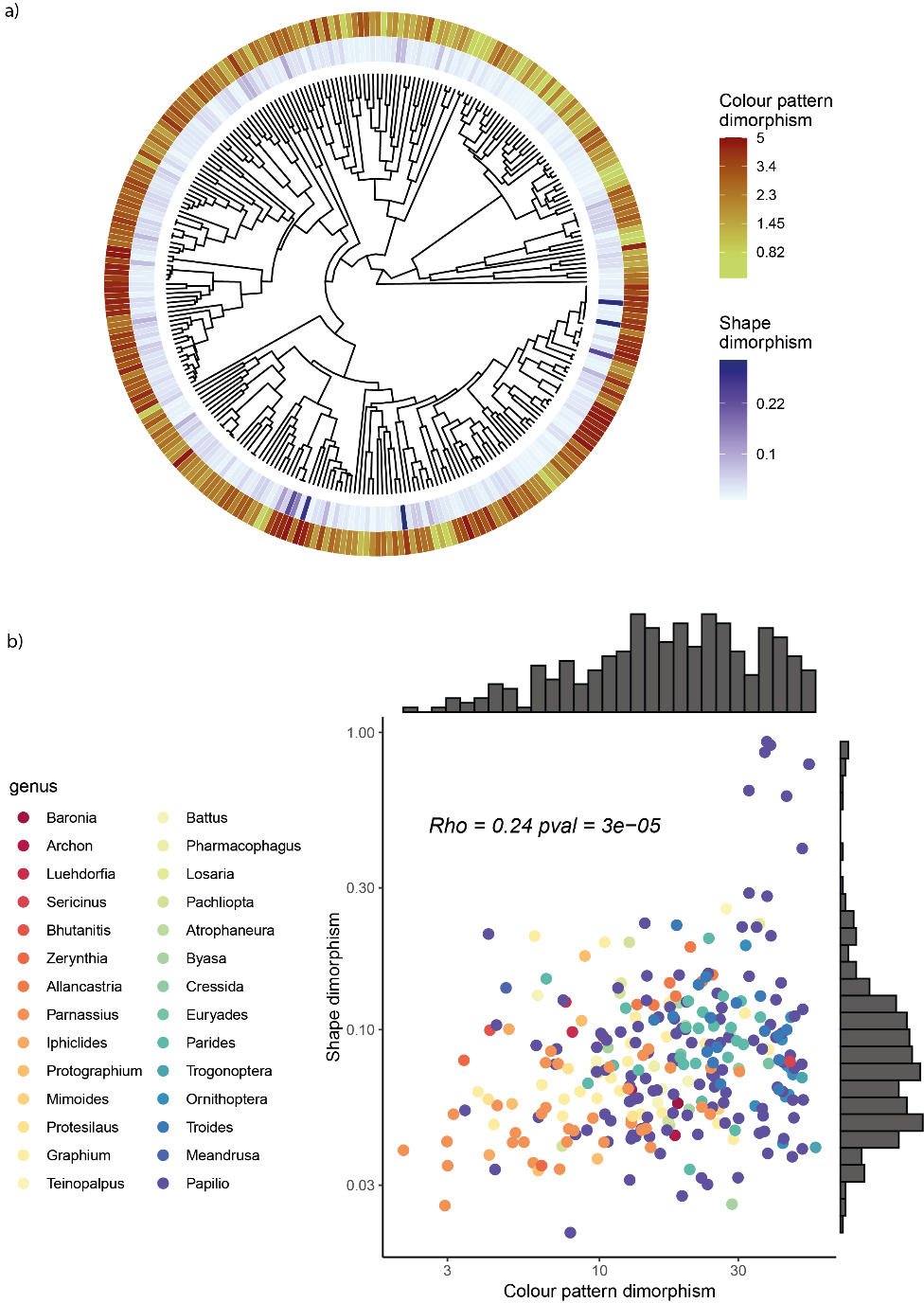


Figure 8. Correlation of shape and ventral colour pattern dimorphism at the macroevolutionary scale. a) Shape and ventral colour pattern dimorphism along the phylogeny. b) Distribution of shape and ventral colour pattern dimorphism and rank correlation (Spearman rho = 0.25, *P*<0.001). The colours correspond to the genus, the x and y axis are log scaled.

## Analysis the association of tail shape and complex spots

We tested whether results differed for eyespots and simple spots. We used the definition of eyespots being approximately circular spots containing several contrasting colours. We separated the spots in our study into simple spots and “complex spots” (that we assimilate to eyespots) using a condition based on colour rather than spot shape because of the often deformed spots due to wing shape changes. We first cropped the images to the spots position for each spot, and segmented the colours on these cropped images using the *recolourize* R package. We then considered that spots with one colour corresponded to simple spots, and spots with more than one colour corresponded to complex spots. Although statistical power is reduced, we obtained results consistent with our main results regarding all spots, with complex spots closer to the wing contours and to the CU2 landmark for longer tailed species.

|  | All species | | Only tailed species | |
| --- | --- | --- | --- | --- |
| **term** | **Estimate** | **P** | **Estimate** | **P** |
| Females, dorsal side | | | | |
| Distance to contours | -0,0306 | 0,0721 | **-0,0403** | **0,0235** |
| Distance to CU2 | **-0,0377** | **0,0574** | -0,0457 | 0,0754 |
| Distance to centroids | **0,0416** | **0,0310** | -0,0227 | 0,2530 |
| Distance to contours:Distance to CU2 |  |  | **-0,0364** | **0,0105** |
| Distance to contours:Distance to centroids |  |  | **0,0488** | **0,0107** |
| Distance to CU2:Distance to centroids |  |  | **0,0623** | **0,0070** |
| Females, ventral side | | | | |
| Distance to contours | 0,0085 | 0,6835 | -0,0188 | 0,3218 |
| Distance to CU2 | -0,0163 | 0,4952 | -0,0112 | 0,6390 |
| Distance to centroids | 0,0047 | 0,8132 |  |  |
| Distance to contours:Distance to CU2 | -0,0244 | 0,1638 | -0,0281 | 0,0764 |
| Distance to contours:Distance to centroids | 0,0285 | 0,0793 |  |  |
| Males, dorsal side | | | | |
| Distance to contours | **-0,0422** | **0,0256** | **-0,0265** | **0,0380** |
| Distance to CU2 | -0,0189 | 0,3527 | -0,0145 | 0,3542 |
| Distance to centroids | -0,0245 | 0,1692 | **-0,0186** | **0,0810** |
| Distance to CU2:Distance to centroids | -0,0250 | 0,1459 |  |  |
| Distance to contours:Distance to CU2 |  |  | **-0,0206** | **0,0555** |
| Males, ventral side | | | | |
| Distance to contours | **-0,0351** | **0,0325** |  |  |
| Distance to CU2 | -0,0324 | 0,0610 | -0,0107 | 0,3651 |
| Distance to centroids | 0,0220 | 0,1842 | 0,0034 | 0,7282 |
| Distance to contours:Distance to centroids | **0,0424** | **0,0017** |  |  |
| Distance to CU2:Distance to centroids |  |  | 0,0121 | 0,1520 |

Table 5: Results of phylogenetic regressions for complex spots only. Red colour depicts negative associations with tail elongation, blue colour depicts positive association with tail elongation.

## Assessing the removal of the tails on colour pattern quantification

## Granularity analysis

To assess whether the removal of the tails could impact the granularity analysis, we compared the energy spectra of tail-removed vs. whole butterflies. We found little differences with a mean relative RMSE (root mean squared error) of ~1.5% of differences for all species, and a mean relative RMSE of ~1.3% when considering only tailed species. Moreover, we tested whether the maximum energy (marking contrast) and pattern size corresponding to maximum energy (marking size) were significantly different between tail-removed and whole butterfly granularity analyses. We found that the marking size did not differ significantly (Wilcoxon, V = 1057, *P* = 0.3693), and that the marking contrast differed significantly (Wilcoxon, V = 3127573, *P* < 2.2e-16). However, the mean difference of maximum energy was 0.009 higher for images with tails, corresponding to a 0.02% change of maximum energy. Overall, tail removal has little effect on the total granularity, but ensures that the quantification of colour pattern descriptors is independent from the quantification of tail shape.


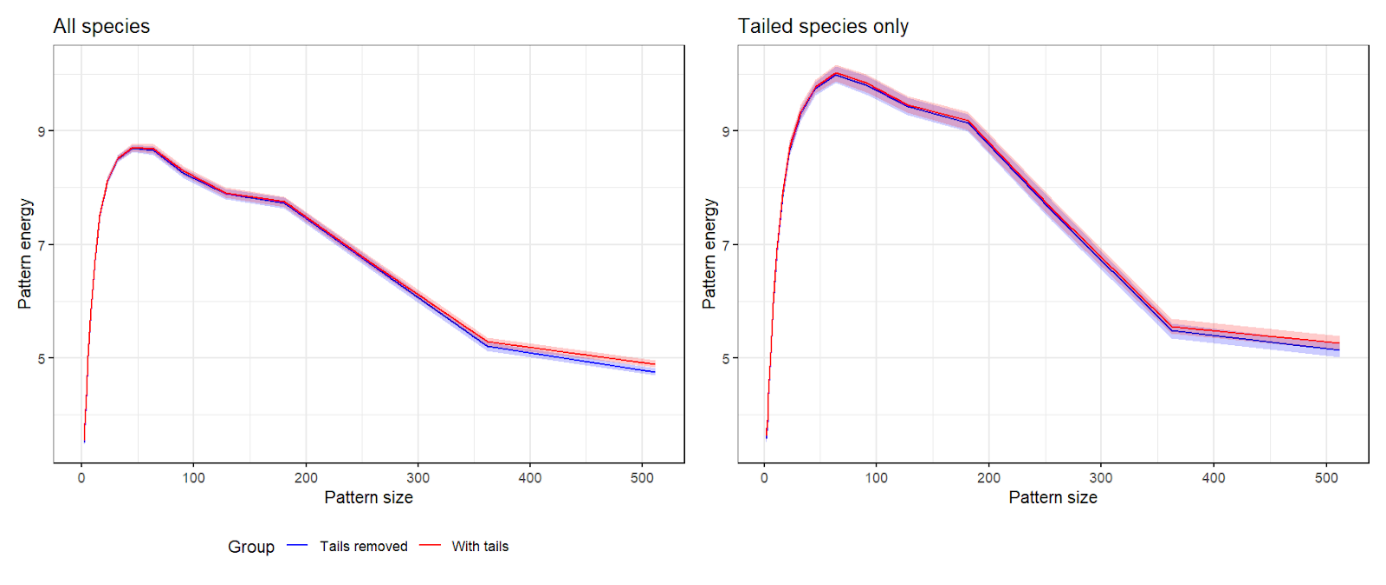


Figure 9: Average spectrum with tails (red) and with tails removed (blue) with 95% confidence interval showing little differences between the two spectra.

## Machine learning embeddings

We re-trained the machine learning model using images of butterflies without removing the tails. We obtained the coordinates for species within the embedding space similarly than in the main results, and re-ran the pPLS analyses. Overall, we found that using the whole butterfly image led to a slightly higher pR-PLS, likely because the tail shape information influences the embedding space organization and thus artificially inflates the correlation between tail shape and colour pattern.

## Clustering on PLS coordinates

We performed a clustering analysis based on the PLS coordinates of the tailed species, using k-means with a silhouette analysis (a measure of within-cluster similarity compared to between-cluster similarity) to find the optimal clustering. We found an optimal number of clusters of 2 for the dorsal and ventral sides of males and females (see figures below), which broadly correspond to the striped/long tailed and spotted/short tailed visual features.


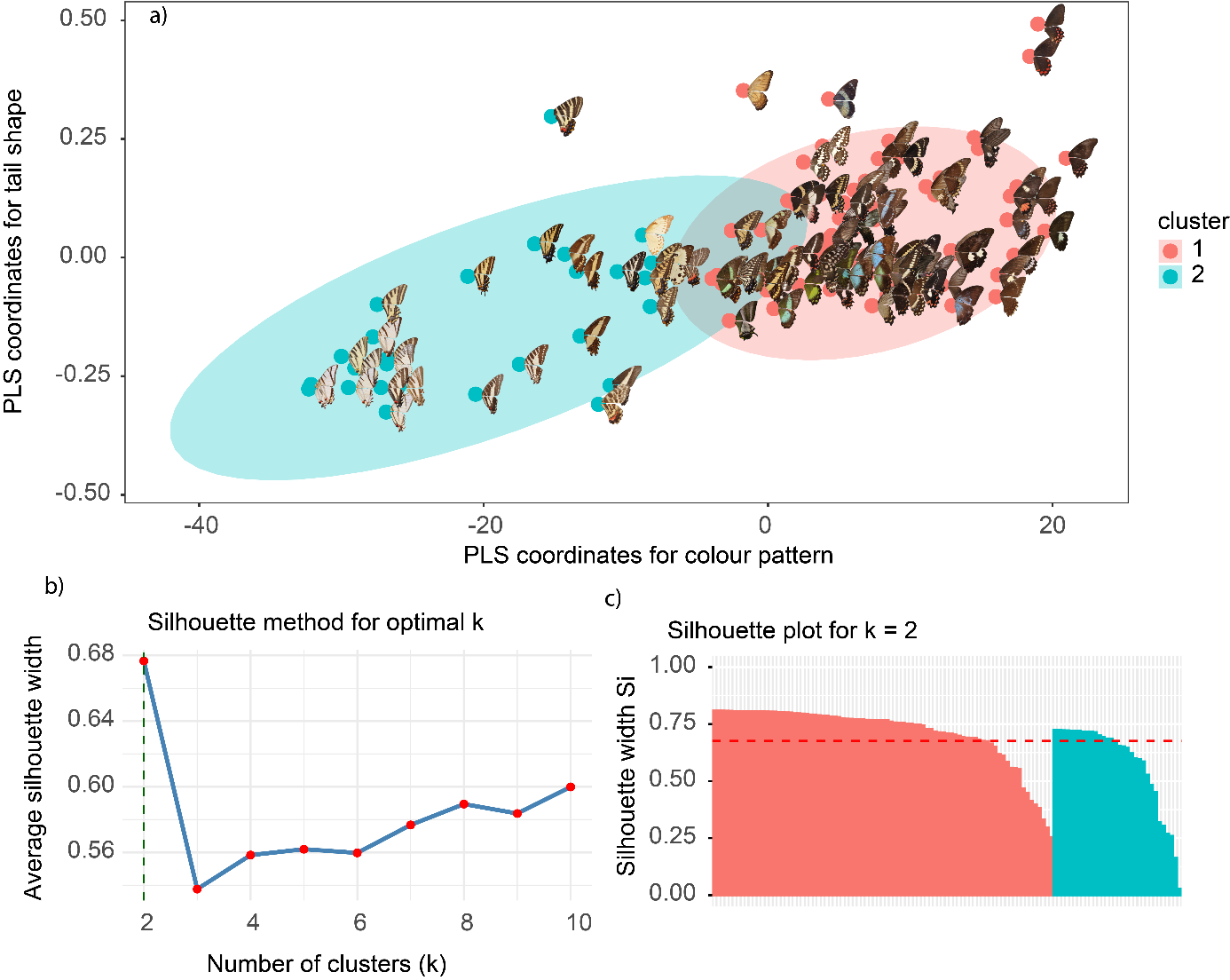


Figure 10: K-means clustering on PLS coordinates shows two main clusters on the dorsal side of females. a) PLS coordinates are coloured depending on their cluster assignments, and the normal data ellipse are superimposed to show cluster distribution in the PLS space. b) The average silhouette score depending on number of clusters shows that the highest score is reached for two clusters. c) Detailed silhouette score for each point of each cluster for two clusters using k-means. The dotted red line denotes the average silhouette score.


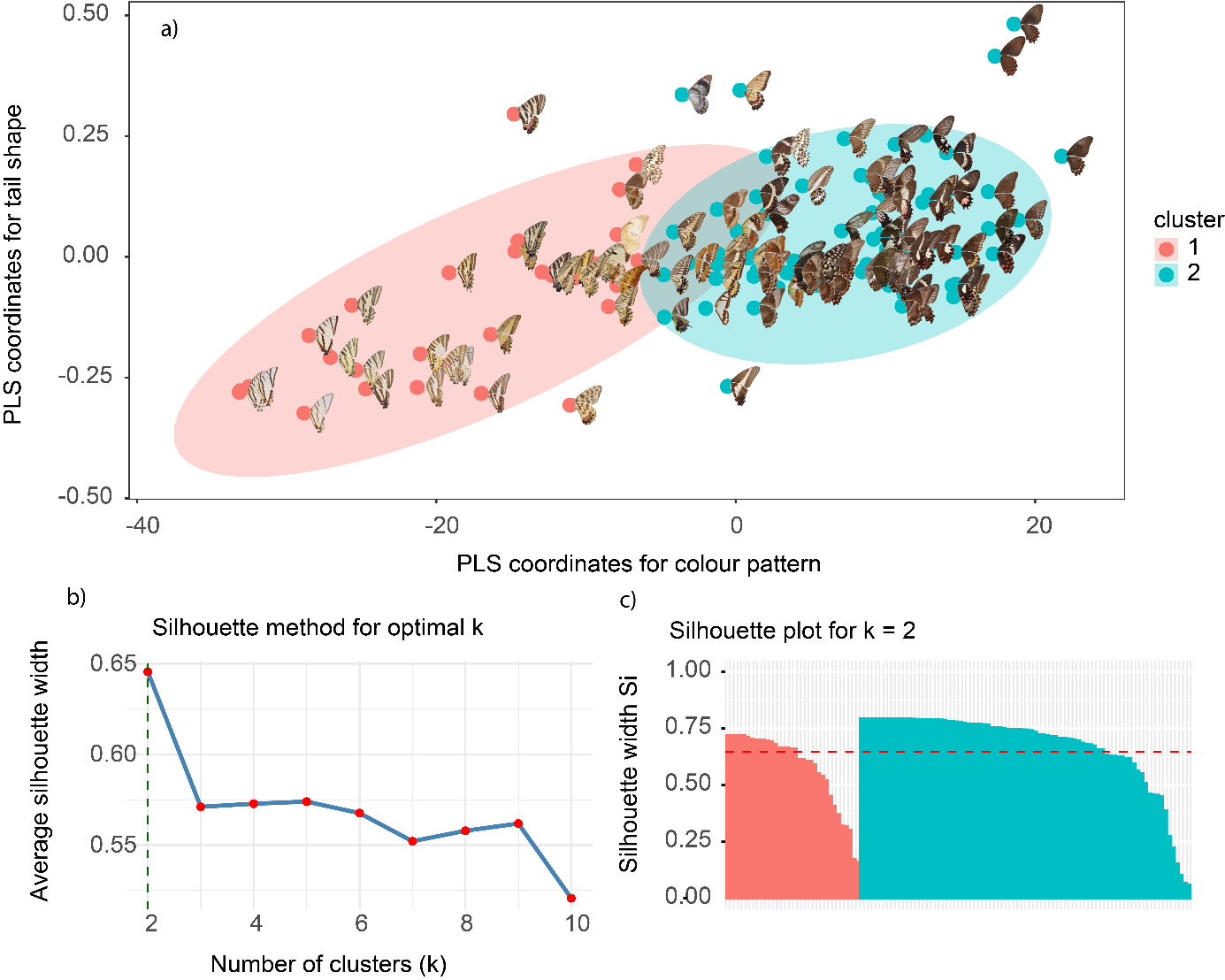


Figure 11: K-means clustering on PLS coordinates shows two main clusters on the ventral side of females. a) PLS coordinates are coloured depending on their cluster assignments, and the normal data ellipse are superimposed to show cluster distribution in the PLS space. b) The average silhouette score depending on number of clusters shows that the highest score is reached for two clusters. c) Detailed silhouette score for each point of each cluster for two clusters using k-means. The dotted red line denotes the average silhouette score.


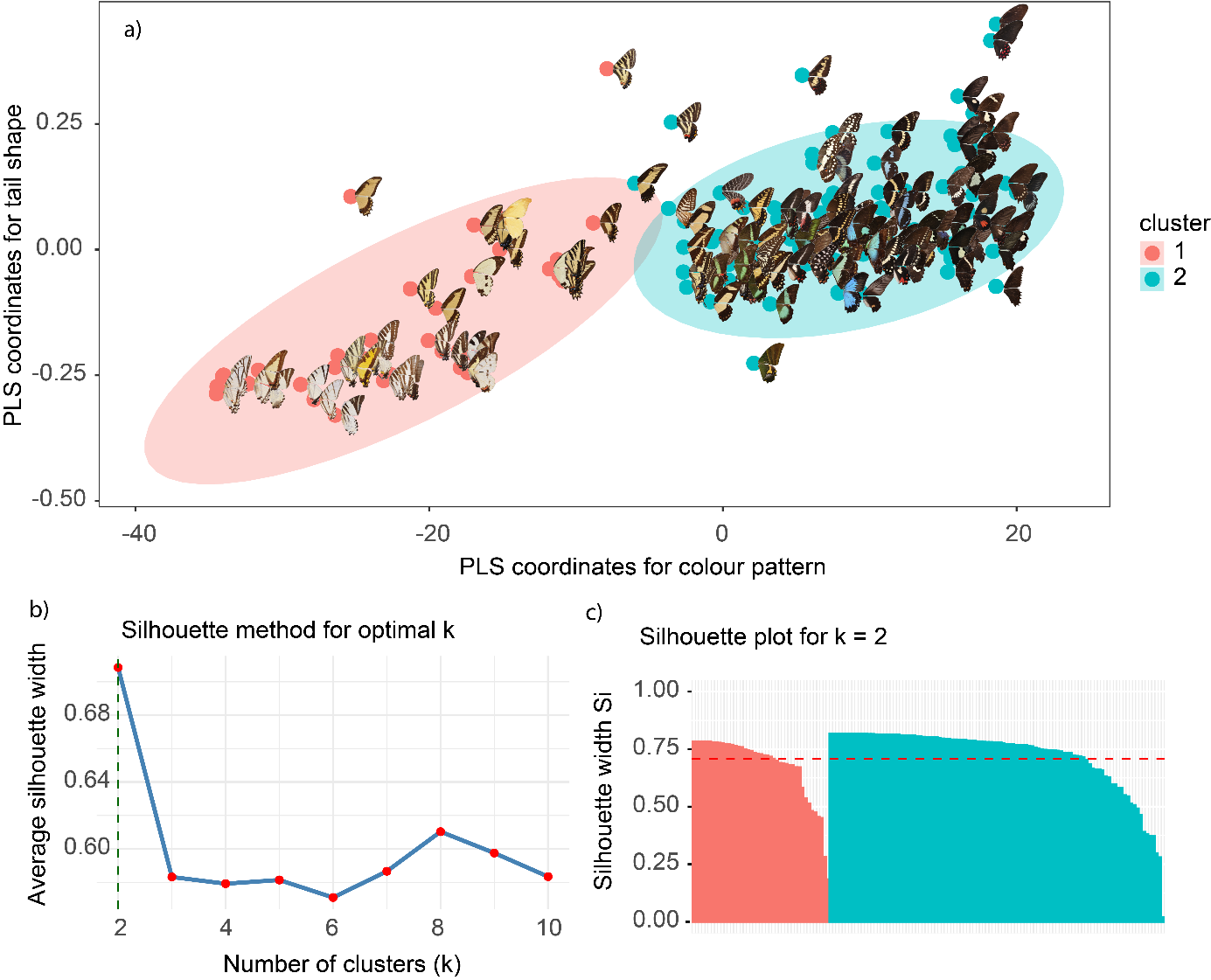


Figure 12: K-means clustering on PLS coordinates shows two main clusters on the dorsal side of males. a) PLS coordinates are coloured depending on their cluster assignments, and the normal data ellipse are superimposed to show cluster distribution in the PLS space. b) The average silhouette score depending on number of clusters shows that the highest score is reached for two clusters. c) Detailed silhouette score for each point of each cluster for two clusters using k-means. The dotted red line denotes the average silhouette score.


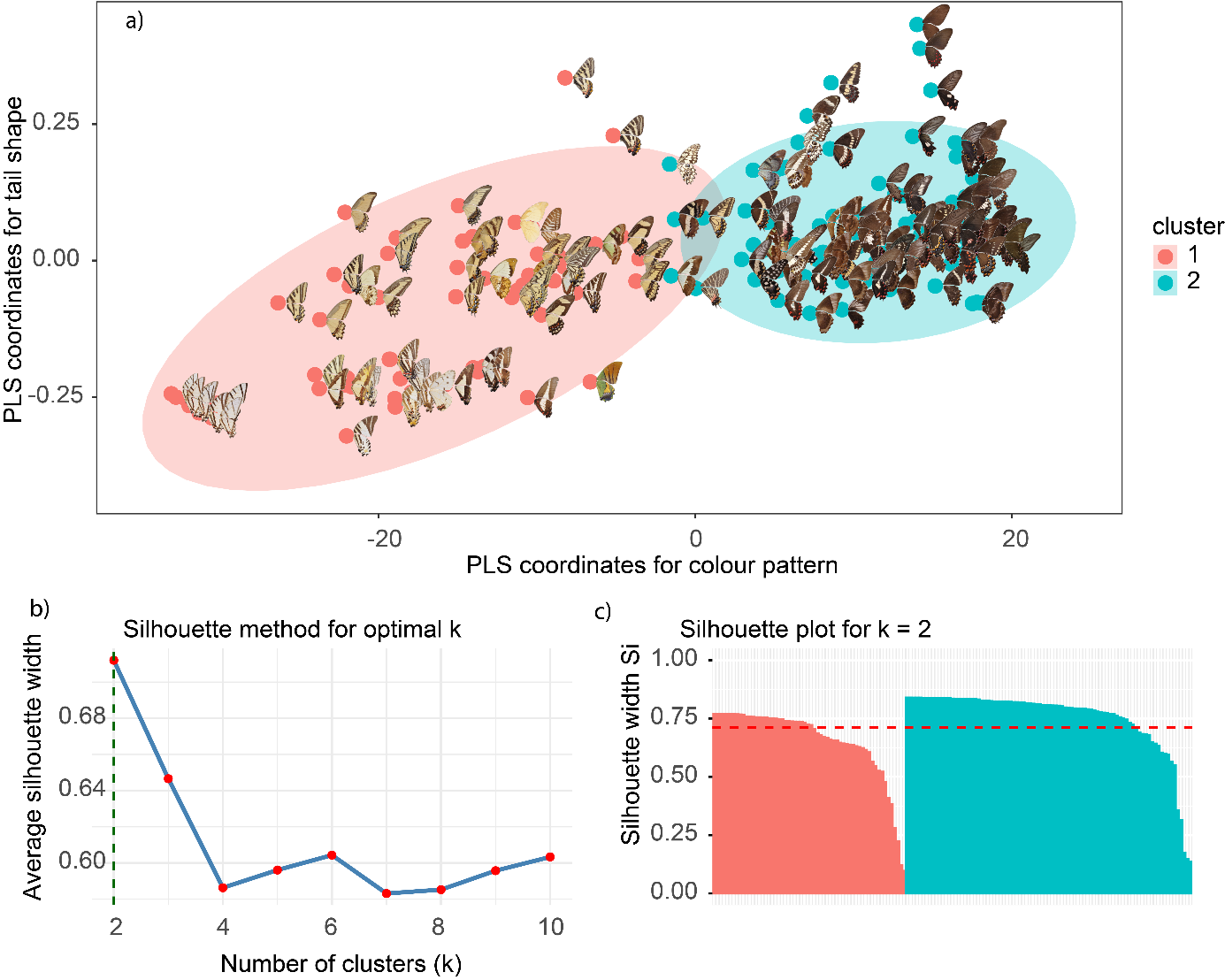


Figure 13: K-means clustering on PLS coordinates shows two main clusters on the ventral side of males. a) PLS coordinates are coloured depending on their cluster assignments, and the normal data ellipse are superimposed to show cluster distribution in the PLS space. b) The average silhouette score depending on number of clusters shows that the highest score is reached for two clusters. c) Detailed silhouette score for each point of each cluster for two clusters using k-means.
